# Self-Healing in Cellulose-Based Materials: From Fundamentals to Future Perspectives

**DOI:** 10.3390/polym18111296

**Published:** 2026-05-25

**Authors:** Bogdan-Marian Tofanica, Elena Ungureanu

**Affiliations:** “Ion Ionescu de la Brad” Iasi University of Life Sciences, 3 Mihail Sadoveanu Alley, 700490 Iasi, Romania; bogdan.tofanica@iuls.ro

**Keywords:** cellulose, cellulose hydrogels, self-healing polymers, bio-based materials, smart materials

## Abstract

Self-healing materials have attracted increasing attention as a strategy to enhance durability, extend service life, and reduce maintenance in advanced material systems. Among these, cellulose-based self-healing materials represent a sophisticated intersection between sustainable macromolecular chemistry and adaptive materials science. This review provides a synthesis of recent advancements in the field, systematically categorizing materials derived from cellulose raw materials. We evaluate the fundamental chemical strategies employed to achieve autonomous repair, distinguishing between extrinsic mechanisms—utilizing cellulose-based micro/nano-capsules to sequester healing agents—and intrinsic mechanisms governed by dynamic covalent chemistry (Schiff-base, boronic ester, Diels–Alder) and supramolecular interactions (hydrogen bonding, metal–ligand coordination, and host–guest assemblies). The analysis highlights how cellulose’s hierarchical structure and abundant surface functionality are leveraged to overcome the traditional trade-off between mechanical toughness and healing efficiency. Particular emphasis is placed on the transition from simple structural hydrogels to sophisticated multifunctional systems. These include ultra-stretchable strain and pressure sensors for e-skin applications, biocompatible and injectable matrices for chronic wound management and stem cell delivery, and advanced anti-freezing eutectogels for performance in extreme environments. Furthermore, we explore the integration of cellulose into traditional sectors, such as self-healing concrete utilizing microbe-induced calcification and smart, eco-friendly coatings for corrosion protection. Finally, we discuss critical challenges, including environmental stability, scalability, and the development of standardized evaluation protocols, providing a roadmap for the next generation of bio-derived, sustainable and intelligent materials.

## 1. Introduction

The global pursuit of sustainable, “green” alternatives to petroleum-derived polymers has repositioned cellulose as the premier candidate for next-generation functional materials [1]. As the most abundant renewable biopolymer on Earth, cellulose is distinguished by its unique hierarchical structure, exceptional mechanical properties, and a highly versatile surface chemistry dominated by reactive hydroxyl groups [2,3]. Despite these advantages, traditional cellulose-based materials are susceptible to mechanical failure and micro-cracking during service, which significantly limits their durability and performance in high-tech applications [4,5].

To address these limitations, the integration of self-healing functionalities has emerged as a transformative strategy. Inspired by biological systems, self-healing materials possess the autonomous ability to repair physical damage and restore structural integrity, thereby extending their service life and reducing maintenance costs [6,7]. Within this field, cellulose is no longer viewed merely as a passive structural filler; it has evolved into a dynamic building block capable of participating in complex, reversible chemical networks [4,8,9].

The development of cellulose-based self-healing materials generally follows two pathways: extrinsic healing, which utilizes cellulose-based micro/nanocapsules to sequester healing agents [10,11,12], and intrinsic healing, which relies on the molecular design of reversible cross-linked networks. The latter is particularly promising, leveraging the rich chemistry of cellulose to establish dynamic covalent bonds—such as Schiff-base, boronate ester, and disulfide linkages [13,14,15,16,17]—as well as supramolecular interactions, including multiple hydrogen bonding and metal–ligand coordination [9,18,19,20].

However, while several excellent reviews over the past five years have explored cellulose-based self-healing materials, the vast majority have restricted their scope to fundamental soft-matter chemistry—primarily focusing on the molecular design of hydrogels and their biomedical applications. It is important to clarify that “self-healing” in the context of cellulose materials is a multiscale phenomenon. The majority of current research is concentrated on intrinsic self-healing hydrogels; however, this review adopts a comprehensive perspective, treating cellulose not merely as a matrix for soft electronics, but as a multifunctional scaffold for repair across all material formats—from nano-scale sensors and biomedical dressings to macro-scale structural composites and sustainable packaging.

Recent research has demonstrated that nanocellulose derivatives, specifically cellulose nanocrystals (CNC), nanofibers (CNF), and TEMPO-oxidized cellulose nanofibrils (TOCNF) can effectively resolve the “stiffness-extensibility” trade-off, providing both structural reinforcement and the segmental mobility required for efficient healing [9,21,22,23]. This synergy has paved the way for highly sophisticated multifunctional systems. In the realm of flexible electronics, self-healing cellulose hydrogels and ionogels are being developed as skin-like sensors capable of monitoring human health in real-time [24,25,26,27]. Simultaneously, in the biomedical sector, these materials serve as injectable, bioactive scaffolds for wound healing and tissue regeneration [28,29,30,31]. Beyond these “soft” applications, cellulose-mediated self-healing is also making significant strides in civil engineering, where it facilitates autonomous crack repair in concrete via internal curing and mineral-producing microbial carriers [32,33,34,35].

This review aims to provide a comprehensive overview of the current landscape of cellulose-based self-healing materials. We explore the transition from raw biomass sources to engineered nanostructures, dissect the underlying chemical mechanisms of repair, and highlight the diverse application domains—from intelligent wearable devices to sustainable infrastructure—that represent the frontier of cellulose science.

To provide a rigorous and in-depth analysis, this review narrows its focus to two distinct chemical paradigms of cellulose in self-healing science. First, and primarily, we deeply investigate cellulose as an active macromolecular participant in soft materials (hydrogels, elastomers, and smart coatings), where its modified hydroxyl groups drive intrinsic repair via dynamic covalent bonds (Schiff-base, boronic esters) and supramolecular assemblies. Secondarily, we contrast this with cellulose’s role as a protective micro-scaffold in structural composites (e.g., cementitious matrices), where it extrinsically encapsulates microbial or chemical healing agents. By bridging these two roles—from molecular-level dynamics to macroscopic encapsulation—and corroborating them with a global patent analysis, this review provides a highly focused roadmap for translating fundamental cellulose chemistry into commercially viable smart materials.

Several excellent reviews over the past five years have explored cellulose-based self-healing materials, yet the vast majority have restricted their scope to fundamental soft-matter chemistry—primarily focusing on the molecular design of hydrogels and their biomedical applications [1,2,4,7,10,23,24,27,30,33]. Consequently, a significant research gap exists regarding the translation of these laboratory-scale chemical breakthroughs into heavy-duty commercial applications. This review distinguishes itself by specifically addressing this translational gap. Rather than merely cataloging recent studies, we systematically bridge the fundamental physical chemistry of cellulose networks (intrinsic dynamic healing) with its macroscopic, industrial-scale deployment (extrinsic healing in protective coatings and civil infrastructure).

Furthermore, to rigorously outline the progress toward industrialization, this review uniquely integrates a comprehensive analysis of the global patent landscape—a crucial metric of commercial viability that is currently absent from the existing literature. By mapping the trajectory from dynamic covalent bonds to patented commercial technologies, this study identifies current technological bottlenecks and unresolved issues in environmental stability, and provides a distinct, commercially grounded perspective on the future of self-healing cellulose platforms.

## 2. Building Blocks: Cellulose Sources and Structures

The design of self-healing materials relies heavily on the structural and chemical characteristics of the cellulose precursor. The transition from raw biomass to high-performance dynamic systems involves the selection of specific cellulose “building blocks,” which can be broadly categorized based on their dimensions, solubility, and biosynthetic origin.

**Figure 1:**
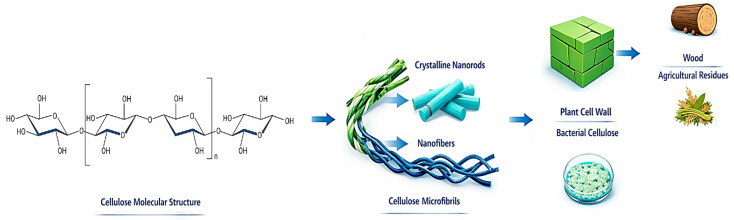
Bottom-up hierarchical architecture of cellulose-based biomass. The left-to-right progression illustrates the organization from the fundamental molecular unit to microfibrillar networks. The right-hand panel distinguishes between plant-derived cellulose (hierarchically organized in cell walls within wood and agricultural residues) and bacterial cellulose (an ultra-pure, 3D nanofibrillar network synthesized via microbial fermentation). Note: Schematic representation for conceptual clarity; not drawn to scale.

From a chemical perspective, the engineering of a self-healing platform must fundamentally begin at the molecular level. As illustrated in Figure 1, the hierarchical architecture of cellulose is fundamentally built upon the linear β(1 → 4)-linked D-glucan macromolecule. At this foundational level, the dense array of surface hydroxyl groups provides the primary reactive sites for chemical functionalizations. Moving up the hierarchy, extensive intra- and intermolecular hydrogen bonding drives these individual polymer chains to self-assemble into highly ordered elementary fibrils, characterized by alternating crystalline and amorphous domains. When chemically or mechanically isolated, these domains yield the essential nanoscale building blocks for dynamic matrices: rigid Cellulose Nanocrystals and long, flexible Cellulose Nanofibers. Ultimately, these nanostructures bundle together to form the micro- and macro-fibers that constitute the complex structural matrices of raw biological sources, the vegetal cell wall, ranging up from the lignocellulosic networks of wood and agricultural residues to the ultra-pure, 3D pellicles produced via bacterial synthesis.

### 2.1. Nanocellulose: CNC, CNF, and TOCNF

Nanocellulose has emerged as the most investigated building block for self-healing systems, owing to its exceptional mechanical modulus (reaching ~150 GPa for crystalline domains), high aspect ratio, and a surface densely populated with reactive hydroxyl groups that facilitate tailored functionalization. In the design of dynamic materials, Cellulose Nanocrystals and Cellulose Nanofibers serve a dual purpose: they act as rigid reinforcing fillers that provide structural integrity while simultaneously participating in the reversible bond network to resolve the classic conflict between mechanical robustness and healing mobility [3,7,9,21]. By modulating the interfacial interactions between these nanostructures and the polymer matrix, researchers can develop materials that dissipate energy through sacrificial bond breakage without permanent structural failure [36,37,38].

Beyond traditional passive reinforcement, nanocellulose acts as a “dynamic bridge” that facilitates autonomous repair across damaged interfaces. A prominent example is the use of TOCNF process that converts primary C6-hydroxyl groups into carboxylate moieties, which serve as active sites for ionic coordination or metal–ligand interactions with cations such as Fe^3+^, Al^3+^, or Zn^2+^ [8,25,26,39,40,41]. These coordination bonds function as reversible cross-links that significantly enhance both the electrical conductivity and the repair efficiency of the network. In many systems, the synergistic interplay between TOCNF and other biopolymers like alginate or gelatin creates a hierarchical interpenetrating network that maintains stability during repeated damage-heal cycles [26,31,41,42,43,44].

The versatility of the nanocellulose surface allows for the introduction of specific functional groups that enable Intrinsic Self-Healing through dynamic covalent chemistry. Aldehyde-functionalized CNCs (a-CNCs) or dialdehyde nanocellulose, produced via periodate oxidation, can react with amine-containing polymers to form reversible Schiff-base (imine) or acylhydrazone bonds [13,16,43,45,46]. Similarly, furan-modified CNFs enable the construction of thermally responsive networks via the Diels–Alder (DA) click reaction with maleimide-functionalized poly(ethylene glycol) or polyurethane [47,48,49,50,51]. These covalent but reversible linkages ensure that the material possesses high fracture strength while remaining capable of rapid recovery upon the application of an external stimulus, such as heat or pH change [47,48,52,53].

The integration of nanocellulose is particularly prevalent in the fabrication of hydrogel-based strain sensors and electronic skins (e-skins). In these applications, nanocellulose serves as a dispersing agent for conductive fillers—such as graphene, carbon nanotubes (CNTs), MXenes, or polyaniline (PANI)—preventing their aggregation and ensuring a stable conductive percolation threshold [4,8,14,15,22,26,41,54,55,56,57,58,59,60,61,62,63,64,65,66]. These nanocellulose-mediated hydrogels exhibit rapid resistive responses and high sensitivity (Gauge Factor), allowing for the real-time monitoring of both large-scale human movements (joint bending) and subtle physiological signals (pulse, respiration, or swallowing) [4,14,32,34,57,58,67,68,69].

Furthermore, nanocellulose is vital for achieving high toughness and durability in advanced elastomers, vitrimers, and protective coatings. By incorporating surface-modified CNCs into siloxane or polyurethane matrices, researchers have developed materials that exhibit low-temperature self-healing (at 5 °C or below) and exceptional fatigue resistance [20,53,70,71,72,73]. The high-density hydrogen bonding and nano-confinement effects provided by nanocellulose enable these composites to retain over 95% of their mechanical properties after multiple repair cycles [9,70,73,74]. This resilience is further extended to environmental stability; for instance, nanocellulose-reinforced eutectogels and organohydrogels utilize the synergistic effect of cellulose and non-evaporating solvents (like glycerol or deep eutectic solvents) to maintain healing ability and conductivity in extreme cold (down to −60 °C) or dry conditions [25,67,68,75,76]. Such multifunctional designs have established nanocellulose as a cornerstone for sustainable, high-performance protective systems, ranging from anti-counterfeiting adhesives to anti-corrosive and anti-fouling coatings [23,77,78,79,80,81,82].

### 2.2. Cellulose Derivatives (CMC, EC, HEC, CA, and MC)

Nanocellulose functions as a dispersed, highly crystalline colloidal reinforcement that effectively serves as the solid structural “skeleton” of the composite, whereas water-soluble cellulose derivatives dissolve at the molecular level to form a true, homogeneous continuous polymer matrix. Because these derivatives exist as individual, solvated polymer chains rather than insoluble nanoparticles, they exhibit high macromolecular mobility. This molecular-level mobility is critical for the diffusion and reshuffling of dynamic bonds across damaged interfaces. Furthermore, their true solubility provides excellent film-forming abilities and established industrial scalability, making them highly effective for processing into dynamic hydrogels, continuous films, or microcapsule walls.

Carboxymethyl Cellulose (CMC) is the most prominent derivative in self-healing research, owing to its abundant carboxymethyl groups (–CH_2_COO^−^), which impart high water solubility and a strong anionic character. CMC is a critical building block for pH-responsive and injectable hydrogels used in biomedical applications, such as diabetic wound healing and tumor therapy [28,42,44,46,83,84,85,86,87,88,89]. Its anionic nature enables robust synergistic interactions with metal ions (Fe^3+^, Al^3+^, or Zn^2+^) and cationic polymers (e.g., chitosan or quaternized cellulose), forming dynamic coordination bonds or polyelectrolyte complexes that facilitate autonomous repair [19,89,90,91,92].

Furthermore, the reactivity of CMC allows for the synthesis of dialdehyde-CMC (DCMC), which serves as a macromolecular crosslinker for Schiff-base networks, enabling rapid self-healing in soft sensors and electronic skins [18,42,84,93,94,95,96,97,98,99,100,101]. In optical and energy-management applications, CMC-based hydrogels provide the necessary transparency and haze required for smart windows and light-diffusing films while maintaining structural self-repairability [102,103].

Other derivatives like Ethyl Cellulose (EC) and Methyl Cellulose (MC) occupy specialized niches, particularly in extrinsic self-healing. Due to its hydrophobic nature and film-forming stability, EC is extensively used as a robust wall material for microcapsules or nanocapsules. These capsules sequester liquid healing agents (like linseed oil or corrosion inhibitors) and release them upon mechanical rupture to repair coating defects [10,12,104,105]. Similarly, MC can be processed via electrospinning into core–shell fibers to create self-healing anti-corrosive layers [106].

Hydroxyethyl Cellulose (HEC), a non-ionic derivative, is particularly favored for constructing high-strength supramolecular hydrogels. Its high solubility and ability to participate in extensive hydrogen bonding with poly(acrylic acid) or polyacrylamide frameworks allow for the creation of tough, conductive networks with excellent self-healing efficiency and mechanical recovery [19,107,108].

For systems requiring higher mechanical modulus or thermoplastic-like behavior, Cellulose Acetate (CA) and Cellulose Propionate (CP) are utilized. These derivatives provide a more rigid matrix suitable for shape-memory materials and vitrimer-like elastomers [96,102,109,110,111]. By grafting flexible side chains or introducing dynamic covalent bonds (such as borate esters or Diels–Alder adducts) into these semi-rigid backbones, researchers can achieve a “dual-stimuli” response where the material first recovers its shape and then heals its internal cracks.

The fundamental advantage of using these cellulose derivatives lies in their ability to provide the necessary macromolecular chain mobility at the crack interface. Unlike highly crystalline native cellulose, the substituted chains in derivatives can diffuse across damaged boundaries (the reptation model), facilitating the reshuffling and re-establishment of dynamic covalent or non-covalent bonds [5,27,46,112,113,114,115,116,117]. This molecular-level mobility is the prerequisite for achieving high healing efficiency and structural longevity in fully bio-based materials.

### 2.3. Bacterial Cellulose (BC)

Bacterial Cellulose (BC) represents a unique class of building blocks synthesized extracellularly by various bacteria, most notably those of the genus *Komagataeibacter* [118,119]. From a chemical and structural standpoint, BC is distinguished by its exceptional chemical purity (the absence of lignin and hemicellulose), a significantly higher degree of polymerization compared to most plant celluloses, and an ultra-fine, ribbon-like nanofibrillar network that forms a highly porous 3D architecture during biosynthesis [120]. These intrinsic features make BC an ideal scaffold for the development of high-performance self-healing systems, particularly where biocompatibility and mechanical toughness are paramount [17,45,121,122].

In the biomedical sector, the 3D network of BC acts as a biomimetic template that closely resembles the natural extracellular matrix (ECM). This structural similarity promotes cell adhesion, migration, and proliferation, which are critical for advanced tissue repair. By incorporating dynamic covalent crosslinks—such as reversible Schiff-base interactions between dialdehyde bacterial cellulose and amine-containing biopolymers like quaternized chitosan—researchers have developed multifunctional wound dressings that are both injectable and rapidly self-healing. These BC-reinforced hydrogels provide a moist healing environment, exceptional water retention, and a robust physical barrier that can autonomously re-establish its integrity following mechanical disruption during bodily movements [17,45,121,122].

The interconnected porosity of the BC network is further leveraged to host conductive nanomaterials for electronic skin (e-skin) and wearable sensors. Because BC nanofibers provide a pre-formed, stable 3D template, they facilitate the organization of conductive fillers like MXene nanosheets or core–shell Liquid Metal (LM) nanodroplets into well-defined electrical pathways [13,60]. In these systems, the BC scaffold provides the necessary indentation resistance and “notch insensitivity,” while the dynamic polymer matrix (e.g., polyacrylic acid or chitosan) enables the actual self-healing of both mechanical and electrical functions. For instance, the synergistic integration of BNC with sulfonated polymers and MXene can lead to ultra-stretchable devices (up to 3200% elongation) that maintain high sensitivity and patternable electroluminescence even after repeated repair cycles [60].

Beyond soft-matter applications, the inherent mechanical strength and high specific surface area of BC are utilized in modern civil engineering. BC acts as an effective “micro-carrier” for mineral-producing microbes (e.g., *Lysinibacillus sphaericus*) within cementitious matrices. Its dense but porous nanofibrillar structure provides a protective environment that maintains the viability of the bacteria against the high-alkaline conditions of concrete. When macroscopic cracks occur (up to 2.5 mm wide), the BC-encapsulated bacteria are activated to facilitate biocalcification, sealing the cracks through the precipitation of calcium carbonate and thereby restoring the mechanical and durability performance of the structure [33].

### 2.4. Fibers and Waste-Derived Cellulose

Aligning with the global shift toward a circular economy and the United Nations Sustainable Development Goals, recent research has focused on obtaining cellulose from agricultural and industrial waste streams. Rather than relying on high-purity wood pulp, scientists are increasingly utilizing raw biomass—such as grape pomace, bamboo pulp, and sisal—to extract high-value nanocellulose building blocks for sustainable self-healing composites [1,112,123,124].

The extraction process itself has become a focal point of green chemistry. For instance, cellulose nanocrystals have been successfully isolated from grape pomace using eco-friendly Deep Eutectic Solvents (DES), demonstrating a low-carbon footprint pathway for producing reinforcing agents for guar gum-based self-healing hydrogels [123]. Similarly, bamboo pulp has been processed via ball milling and physical blending to create multi-responsive, self-healing hydrogels [112]. Sisal-derived nanocellulose crystals (NCCs), when coated with bio-inspired tannic acid (TA), have proven highly effective in constructing ionically conductive hydrogels that maintain excellent sensitivity and sensing stability for wearable human-motion monitors [124].

In the field of civil engineering, macro-scale and micro-scale cellulose fibers are employed as low-cost, multi-functional carriers to address the inherent brittleness of cementitious materials. Alkali-treated microcellulose fibers serve as “smart scaffolds” for mineral-producing microbes (e.g., *Bacillus subtilis*), protecting the bacteria during the high-stress mixing process of mortar and concrete [32,125,126]. These fibers act as physical “bridges” across micro-cracks, facilitating the uniform precipitation of calcium carbonate (biocalcification) and increasing the availability of bacteria in the damaged region.

Beyond microbial delivery, cellulose fibers function as internal water reservoirs. In Ultra-High Performance Concrete (UHPC), cellulose nanofibers (CNFs) and nanocrystals (CNCs) promote “internal curing” by releasing stored moisture to hydrate unreacted cement particles within the crack zone, thereby sealing the cracks and restoring the material’s water tightness and mechanical stiffness [34,35,125]. This is particularly critical for infrastructure exposed to aggressive environments, such as chlorides and geothermal waters.

The integration of cellulose fibers with other natural polymers has led to the development of fully bio-based, self-repairing green composites. For example, microfibrillated cellulose (MFC) is combined with soy protein isolate (SPI) to create “all-green” structural materials. In these systems, the inherent high strength of the cellulose fibrils and their extensive hydrogen bonding with the protein resin ensure mechanical robustness, while incorporated microcapsules provide the extrinsic self-healing mechanism required to bridge fracture surfaces [127].

Furthermore, cellulose nanofibers are being integrated into novel bio-based copolymacrolactones (such as poly(ethylene brassylate-co-squaric acid)) to create gels that exhibit both hydrophobic behavior and self-healing ability [128]. These waste-derived systems not only reduce reliance on petroleum-based polymers but also provide a promising strategy for developing durable, repairable materials for low-stress applications like agricultural wrapping, cushioning, and sustainable electronics [3,96,128].

Having established the hierarchical and chemical diversity of cellulose matrices—ranging from nanoscale fibrils to functional derivatives—it becomes evident that their potential for self-healing is fundamentally determined by their chemical availability. The structural characteristics discussed in this section serve as the prerequisite for the activation of dynamic repair pathways. Specifically, the availability of surface functional groups and the inherent mobility of the polymer chains dictate which dynamic bonding strategies can be implemented. This leads us to the fundamental chemical principles governing damage recovery, where these cellulose matrices are engineered to host the dynamic covalent and supramolecular networks detailed in the subsequent section.

## 3. Self-Healing Mechanisms: The Chemistry of Reversible Networks

**Figure 2:**
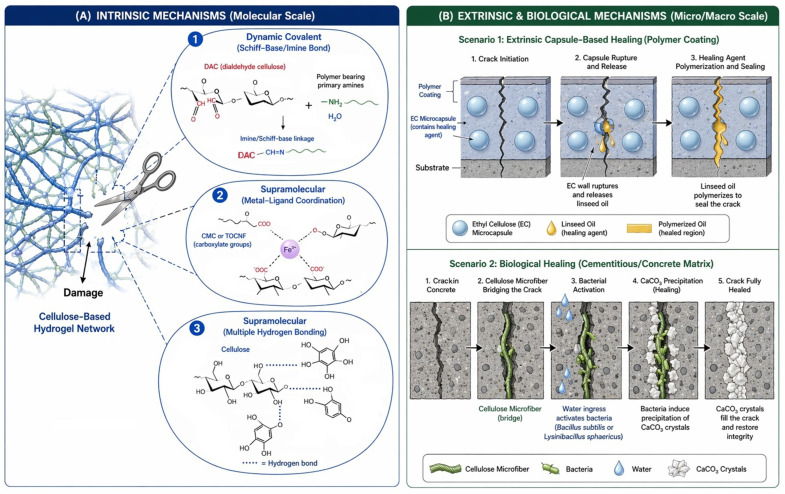
Fundamental self-healing mechanisms in cellulose-based systems. (**A**) Intrinsic molecular repair driven by dynamic covalent chemistry (e.g., Schiff-base formation between dialdehyde cellulose and amines) and supramolecular interactions, including metal–ligand coordination (Fe^3+^/Zn^2+^) and dense multiple hydrogen-bonding networks. (**B**) Extrinsic repair strategies utilizing ethyl cellulose microcapsules for the targeted release of healing agents in coatings, and cellulose-mediated microbe-induced calcium carbonate precipitation (MICP) for autonomous crack sealing in cementitious matrices.

The core of self-healing science lies in the “dynamic equilibrium” established within the material’s molecular architecture. For cellulose, which is inherently rigid due to its extensive intra- and intermolecular hydrogen bond network, achieving self-repair requires chemical modification to introduce functional groups capable of “molecular mending.” As illustrated in Figure 2, the strategies to overcome this rigidity and impart self-healing capabilities can be broadly divided into intrinsic and extrinsic mechanisms. Intrinsic healing requires the strategic implementation of dynamic covalent chemistry and supramolecular interactions, allowing the high-modulus cellulose chains to participate in reversible bond-breaking and bond-reforming processes at the molecular level. Conversely, extrinsic and biological healing bypasses the need for molecular mobility by utilizing cellulose as a protective micro-reservoir or scaffold. In these systems, cellulose capsules or fibers sequester liquid healing agents or mineral-producing bacteria, which are autonomously released upon mechanical rupture to physically seal macroscopic cracks.

### 3.1. Dynamic Covalent Bonds (DCC)

Dynamic covalent bonds (DCBs) represent a sophisticated class of crosslinks that bridge the gap between the robustness of traditional covalent architectures and the reversibility inherent in supramolecular interactions. By integrating DCBs into cellulose-based systems, researchers can develop materials that maintain high mechanical strength while permitting the structural reconfiguration required for damage recovery.

A predominant strategy in this field involves the utilization of Schiff-base chemistry, encompassing both imine and hydrazone linkages, which remains the most widely investigated dynamic covalent link in cellulose science. This mechanism typically proceeds through the condensation of aldehyde groups—frequently introduced via the periodate oxidation of cellulose or nanocellulose to form dialdehyde derivatives—with various amine or hydrazide moieties [13,16,43,45,46,121,122]. Due to their inherent reversibility and sensitivity to environmental stimuli such as pH or moisture, these linkages are exceptionally well-suited for the design of injectable biomedical hydrogels, adhesive wound dressings, and skin-like sensors [15,28,29,42,44,83,84,89,93,99,101,129,130,131,132,133]. Furthermore, the adoption of bottlebrush copolymer architectures and the deployment of aromatic Schiff-base motifs have been shown to collaboratively enhance macromolecular mobility, frequently resulting in healing efficiencies that exceed 95% [42,45,113,122].

In parallel, boronic ester bonds have gained significant attention for their rapid healing kinetics and stimulus-responsive behavior. These bonds are formed through the complexation of phenylboronic acid—strategically grafted onto cellulose or carboxymethyl cellulose backbones—with 1,2- or 1,3-diols, which may be provided by the native cellulose chains or through the incorporation of poly(vinyl alcohol) (PVA) [58,66,95,97,100,107,124,132,133,134,135]. The hallmark of boronic ester-based networks is their exceptionally fast self-healing capability, often occurring in under 30 s without external intervention. Consequently, these bonds frequently serve as “dynamic bridges” in the construction of ionically conductive hydrogel sensors and supercapacitors, where they ensure the simultaneous restoration of mechanical integrity and electrical pathways [14,37,39,40,41,56,57,58,61,64].

For applications requiring high-modulus materials or industrial-grade durability, the Diels–Alder (DA) click reaction offers a robust, thermally reversible alternative. This [42] cycloaddition involves the interaction between furan groups, grafted onto cellulose or nanocellulose, and maleimide-terminated functional polymers. The resulting DA adducts allow for the repeated recycling and healing of high-strength elastomers and vitrimers, effectively bridging the gap between bio-based sustainability and high-performance engineering requirements [47,48,49,50]. Similarly, disulfide bonds leverage redox-responsive and light-triggered reshuffling to enable the construction of “ultrarobust” supramolecular materials [51,72,136]. In these hybrid systems, cellulose nanofibers, which provide a dense hydroxylated surface, interact with disulfide-containing waterborne polyurethanes. This integration facilitates rapid self-healing through a synergistic interplay of multiple dynamic bonds, ensuring that the resulting nanocomposites possess both the stiffness associated with cellulose and the adaptive resilience of a dynamic covalent network [15,79,136].

### 3.2. Supramolecular (Physical) Interactions

Supramolecular chemistry leverages non-covalent forces to mediate the re-bonding of fractured surfaces, an approach that typically requires significantly lower activation energies than dynamic covalent pathways and facilitates autonomous healing at room temperature. Native cellulose is fundamentally defined by its dense internal hydrogen-bonding network, while a “dynamic” hydrogen-bonding environment is created by strategically incorporating additives such as tannic acid (TA) or poly(vinyl alcohol) [4,9,18,26,56,59,61,73,77,98,103,132,137]. These secondary components introduce a hierarchy of sacrificial bond networks that effectively dissipate mechanical energy through reversible bond rupture and reformation. This mechanism affords cellulose-based hydrogels and films exceptional toughness, fatigue resistance, and shape-memory properties, as the reversible physical crosslinks can be repeatedly triggered to restore structural integrity after significant deformation [8,15,20,36,65,96,107,114,129,138,139,140].

Furthermore, the coordination chemistry between multi-valent metal ions—most notably Fe^3+^, Al^3+^, or Zn^2+^—and carboxylated cellulose derivatives, such as CMC or TOCNF, represents an essential strategy for developing high-performance hybrid materials [18,19,21,25,54,58,67,76,86,90,91,92,98,108,117,124,132,135,141,142,143]. The resulting metal–ligand coordination networks provide a synergistic balance between mechanical robustness and ionic conductivity, facilitating the fabrication of multifunctional platforms. The presence of these metal centers not only enhances the tensile and compressive strength of the matrix through high-density crosslinking but also provides the inherent stimuli-responsiveness required for advanced sensing devices, character recognition boards, and smart, anti-corrosive coatings [19,58,135].

Complementing these interactions, host–guest molecular recognition offers a more precise level of control over the assembly and disassembly processes of cellulose architectures. The specific recognition between macrocyclic hosts, such as β-cyclodextrin, and designated guest molecules like adamantane or azobenzene, allows for the engineering of hydrogels with highly tunable viscoelastic properties. These host–guest complexes are frequently integrated alongside other dynamic covalent crosslinks to formulate injectable, self-healing reservoirs capable of controlled and site-specific drug release, thereby broadening the utility of cellulose in regenerative medicine and pharmaceutical engineering [130,131]. Collectively, these supramolecular strategies transform the rigid cellulose backbone into an adaptive, “living” network capable of autonomous response to environmental and mechanical stimuli.

### 3.3. Extrinsic and Biological Mechanisms

A distinct shift from molecular-level intrinsic repair is found in extrinsic self-healing methodologies, which frequently emulate the biological “clotting” response through the sequestration of active healing agents within specialized cellulose-based micro-reservoirs. The utilization of Ethyl Cellulose (EC) as a wall material for microcapsules and nanocapsules is particularly advantageous in this context, owing to its hydrophobic character, film-forming stability, and relative chemical inertness toward diverse core materials [10,12,104,105]. Beyond spherical capsules, core–shell fibers fabricated via electrostatic spinning—often utilizing methyl cellulose—offer an alternative architecture for integrating healing functionalities directly into a polymer matrix [106]. Upon the occurrence of mechanical damage, these encapsulated reservoirs or fibers are ruptured, facilitating the autonomous release of liquid agents such as linseed oil, dodecylamine, or other specialized corrosion inhibitors [81,82,144,145,146]. Once released, these agents undergo secondary reactions, such as oxidative polymerization or film-forming adsorption, to effectively fill and seal the crack voids, thereby restoring the material’s barrier properties and preventing further degradation of the underlying substrate [10,116,127].

In a pioneering intersection of biotechnology and civil engineering, cellulose is also employed as a “smart carrier” in the context of Microbe-Induced Calcium Carbonate Precipitation (MICP). This biological self-healing strategy addresses the perennial challenge of macroscopic cracking in cementitious materials by utilizing alkaliphilic bacteria, such as *Bacillus subtilis* or *Lysinibacillus sphaericus*, to facilitate autonomous repair [32,33,126]. Within the dense and highly alkaline environment of concrete, the cellulose matrix acts as a protective habitat, maintaining the viability of the mineral-producing microbes while simultaneously serving as a water reservoir that aids in internal curing. As cracks propagate and allow for the ingress of moisture and oxygen, the encapsulated bacteria are activated to catalyze the hydrolysis of urea, leading to the localized precipitation of calcium carbonate (CaCO_3_). These mineral deposits effectively bridge and seal cracks up to several millimeters in width, thereby restoring the structural durability and longevity of the infrastructure through controlled biological mineralization [33,126]. This synergy between the protective properties of cellulose and the metabolic activity of bacteria represents a significant breakthrough in the development of low-maintenance, sustainable construction materials.

### 3.4. Comparative Analysis: Strengths, Limitations, and the Stiffness-Extensibility Trade-Off

Both intrinsic and extrinsic self-healing mechanisms offer distinct advantages; however, their practical application is limited by significant fundamental trade-offs. The most critical limitation in cellulose-based intrinsic self-healing is the stiffness-extensibility paradox. Because intrinsic healing relies on polymer chain mobility (reptation) and the rapid reshuffling of dynamic bonds across a fractured interface, the polymer network must remain relatively “soft” or highly solvated. Consequently, hydrogels utilizing dynamic covalent bonds (like boronate esters or Schiff-base) often exhibit extraordinary healing efficiencies (>95%) and rapid kinetics (under 60 s), but they inherently lack the high mechanical modulus required for load-bearing applications [58,95,97,107,124,134,135].

Conversely, extrinsic systems (e.g., ethyl cellulose microcapsules) bypass this paradox. They can be embedded within rigid, high-modulus matrices like polyurethane coatings or cement, maintaining excellent structural robustness. However, extrinsic healing is fundamentally limited by its “one-time use” nature; once a capsule is ruptured and the localized healing agent is depleted, the material cannot repair secondary damage at the exact same location. In contrast, intrinsic systems allow for multiple, repeated healing cycles, though they often suffer from fatigue resistance issues, where cumulative micro-structural deformations lead to signal drift in sensors after repeated loading [10,12,104,105,106].

Environmental sensitivity remains another significant vulnerability for intrinsic networks. Because dynamic covalent bonds and hydrogen networks rely heavily on hydration, these materials are highly susceptible to dehydration in arid environments or freezing at sub-zero temperatures, which immediately halts the healing process. Although the transition to organohydrogels and eutectogels using deep eutectic solvents has mitigated this issue, new challenges have emerged regarding the toxicity and long-term stability of these solvents [25,67,68,75,76].

Finally, regarding scalability and industrial feasibility, a stark contrast exists between the two approaches. The complex, multi-step synthesis required to create multi-dynamic intrinsic networks—often involving expensive catalysts, rare metal ions, and precise pH control—makes them economically challenging to scale for commodity markets. This is strongly reflected in the commercial sector, where industrial patents overwhelmingly favor the cost-effective, scalable production of extrinsic microcapsules to protect heavy-duty infrastructure over the delicate synthesis of intrinsic soft matter. Ultimately, the successful commercialization of these technologies will require hybrid designs that balance the regenerative longevity of dynamic bonds with the environmental resilience and scalability of extrinsic encapsulation.

The chemical principles of damage recovery, mediated by the dynamic covalent and supramolecular interactions defined above, are not abstract concepts; their efficacy is entirely dependent on the material’s architectural format. The viscoelastic environment required for bond reshuffling is directly shaped by the material’s physical state—whether it is a highly hydrated hydrogel, a thin-film protective coating, or a structural composite. To understand how these repair mechanisms translate into real-world performance, we must now examine the specific material architectures—the gels, films, adhesives, and composites—that facilitate the manifestation of these self-healing behaviors in practical, operational environment.

## 4. Material Formats and Structural Architectures

The practical implementation of self-healing functionalities is heavily dictated by the physical format of the material, ranging from highly hydrated 3D networks to robust structural composites.

### 4.1. Gel Architectures: Hydrogels, Organohydrogels, and Eutectogels

Hydrogels and their solvent-modified derivatives represent the most pervasive and versatile material formats in cellulose self-healing research. These three-dimensional macromolecular networks provide a unique viscoelastic environment that is essential for facilitating molecular chain mobility and the efficient reshuffling of dynamic bonds across fractured interfaces [1,2,5]. The highly hydrated nature of these systems allows for the plasticization of the cellulose backbone, enabling the reptation of polymer chains which is a prerequisite for autonomous repair. For instance, self-healing cellulose hydrogels (SHCHs) are frequently engineered through the precise molecular design of complexation between phenylboronic acid-grafted carboxymethyl cellulose and poly(vinyl alcohol). The resulting dynamic boronate ester linkages create a pH-responsive, reversible network that yields biocompatible and biodegradable matrices suitable for advanced drug delivery, 3D cell culture, and tissue engineering [46,87,100].

To overcome the inherent limitations of traditional hydrogels—namely their susceptibility to freezing at sub-zero temperatures and dehydration in ambient conditions—researchers have increasingly turned to organohydrogels and eutectogels. By replacing a significant portion of the aqueous phase with non-evaporating, hygroscopic co-solvents such as glycerol, ethylene glycol, or dimethyl sulfoxide (DMSO), or by utilizing Deep Eutectic Solvents (DES) as the continuous phase, the structural and functional stability of the material is drastically enhanced [69,129,140]. These advanced gel systems are often reinforced with nanocellulose (CNF or CNC) to improve structural integrity and are further cross-linked via ionic coordination with multi-valent metal ions like Zn^2+^ or Al^3+^ [75,135,140]. The presence of these ions, combined with the suppressed crystallization of water molecules within the DES or organo-solvent framework, endows the materials with remarkable anti-freezing properties and ultra-stretchability—reaching values as high as 4086% elongation at break. Such gels maintain their intrinsic self-healing capabilities and ionic conductivity at extreme temperatures as low as −80 °C, ensuring reliability for electronics used in alpine or polar regions.

In the domain of wearable electronics and human–machine interfaces, nanocellulose-mediated conductive hydrogels have become a cornerstone for high-performance device fabrication. Within these architectures, nanocellulose (specifically CNC or TOCNF) performs a dual function: it serves as a structural reinforcement and as a potent dispersing agent that prevents the aggregation of conductive fillers such as carbon nanotubes (CNTs), graphene, or MXene nanosheets [8,41,124,133]. This ensures the formation of a stable, uniform conductive percolation threshold throughout the gel matrix. The resulting sensors act as high-sensitivity strain and pressure transducers, characterized by rapid response times (often <200 ms) and high gauge factors, enabling the precise monitoring of both large-scale human movements—such as joint bending—and subtle physiological signals, including pulse, vocal cord vibration, and respiration [8,38,41,63,124,133,134]. By leveraging the synergistic interplay between the hierarchical cellulose network and dynamic covalent or non-covalent interactions, these multifunctional gels offer a sustainable and resilient platform for next-generation bio-electronics [38,41,134].

### 4.2. Protective Coatings and Functional Thin Films

Cellulose-based coatings and thin films extend the utility of bio-derived materials into the highly demanding realms of surface protection, anti-corrosion, and advanced light management. Unlike bulk gels, these thin-film architectures must maintain high transparency and precise barrier properties while hosting dynamic repair mechanisms. A notable example of high-performance light management is the development of composite films prepared from microfibrillated cellulose (MFC) and benzoxazine resins (BR) using a straightforward calendering-like strategy [102]. These films, comprising 70 wt% MFC, achieve a remarkable tensile strength of 142.99 MPa and utilize the adjustable thermal-curing process of BR to enable on-demand self-healing via simple heating (e.g., with a hair dryer). Beyond mechanical repair, these films offer critical health-centric functionalities, including 98.65% ultraviolet (UV) blocking and 68.85% blue-light blocking, making them ideal candidates for energy-efficient building envelopes. Similarly, the reconfiguration of cellulose into “bifunctional dynamers” through a crystalline-to-amorphous transformation—such as in dialdehyde hydroxypropyl cellulose (DAHPC) systems—allows for the creation of ionogel films that harmonize fracture strengths above 4.2 MPa with autonomous self-repair efficiencies exceeding 96% [103].

In the field of extrinsic corrosion protection, cellulose provides a versatile platform for the encapsulation and controlled release of active healing agents. Recent advancements have focused on CNC-stabilized Pickering emulsions and ethyl cellulose (EC) microcapsules to sequester linseed oil (LO) or specialized inhibitors. In waterborne polyurethane (WPU) systems, CNCs act as sustainable emulsifiers to stabilize LO droplets; upon mechanical injury, the capsules rupture, and the released LO undergoes oxidative polymerization—facilitated by driers—to seal surface scratches and restore anti-corrosive integrity [81,82,147]. Research indicates that ethyl cellulose nanocapsules offer significantly higher corrosion resistance and healing efficiency compared to their micro-scale counterparts when embedded in polyurethane coatings [12]. Furthermore, one-pot assembly strategies have led to the creation of cellulose-silica hybrid microcapsules grafted with UV absorbers and hydrophobic reagents. These coatings not only provide thermal insulation but also exhibit self-repairable superhydrophobicity and UV protection even after multiple chemical or mechanical abrading cycles [148].

The development of “ultrarobust” and self-reporting nanocomposites represents a significant frontier in smart coating technology. By synergistically combining flexible disulfide and urea bonds within a waterborne polyurethane (WPU) matrix reinforced with hydroxyl-rich cellulose nanofibers (CNF), researchers have fabricated films with extraordinary tensile strengths (up to 54.4 MPa) and toughness (255.6 MJ/m^3^) [136]. These materials leverage a strain-induced reversible network under nano-confinement effects to achieve a self-healing efficiency of 92.7%. Crucially, these advanced films exhibit photoluminescence under 365 nm UV irradiation, enabling the visual detection of micro-cracks and serving as a secure platform for anti-counterfeiting applications in clothing and high-value packaging [50,136]. Other vitrimer-like systems utilize cellulose-functionalized halloysite nanotubes (HNT-C) in epoxy coatings to facilitate healing through transesterification reactions, significantly reducing the chain relaxation time and enhancing the coating’s toughness [149].

Finally, the application of cellulose thin films has expanded into extreme environmental protection, including marine antifouling. Double-network (DN) hydrogel coatings reinforced with carboxylated CNF and MXene have demonstrated ultra-high toughness (16,050 kJ/m^3^) and the ability to maintain anti-algal and anti-protein adhesion for over six months in actual marine field tests [23]. These films utilize the high aspect ratio of multi-branched cellulose nanocrystals (MCNCs) or nanofibers to form dense percolation networks that dissipate energy through reversible hydrogen bonding and dynamic covalent reshuffling [23,73]. Whether through rapid room-temperature repair (within 12 s in some CNC/acrylate systems) or photothermal-triggered healing, these cellulose-based architectures provide a sustainable and resilient alternative for the next generation of smart surface technologies [22,23,73].

### 4.3. Self-Healing Adhesives: From Industrial Strength to Bio-Interfacial Design

The development of cellulose-based self-healing adhesives addresses a critical industrial and medical demand for bonding agents that combine high-strength adhesion with the capacity for structural recovery and reusability. By leveraging the abundant surface hydroxyl groups of cellulose for physical or chemical modification, researchers have moved beyond traditional thermosetting resins toward dynamic, “living” adhesive interfaces.

A prominent strategy for industrial-grade sustainability involves the physical blending of cellulose nanocrystals with gallic acid (GA) and poly(N-vinylpyrrolidone) (PVP) to create a supramolecular network driven by a dense hydrogen-bonding matrix [77]. These CNC/GA/PVP adhesives exhibit exceptional lap shear strength—reaching 4.91 MPa on glass, 4.39 MPa on wood, and 3.94 MPa on aluminum—while maintaining high electrical conductivity. Crucially, these systems demonstrate water-triggered re-adhesion, where the simple application of moisture facilitates the re-occurrence of dynamic hydrogen bonds, allowing for repeated reuse without significant loss of bonding integrity.

Other industrial approaches utilize Schiff-base chemistry combined with Reversible Addition-Fragmentation Chain Transfer (RAFT) polymerization to create ethyl cellulose-based graft copolymers. By adjusting the ratio of vanillin and fatty acid moieties, the glass transition temperature and crosslinking density can be fine-tuned to balance high shear strength (up to 1.31 MPa) with a self-healing efficiency of approximately 98.7%. This molecular engineering ensures that the “bottlebrush” structure of the copolymers facilitates the chain mobility necessary for rapid interface reshuffling [113,114].

In the biomedical sector, the focus shifts to tissue adhesion and atraumatic removal. Inspired by the adhesive proteins of marine mussels, researchers have grafted catechol moieties (such as dopamine or tannic acid) onto carboxymethyl cellulose or chitosan backbones to create hydrogel adhesives with robust wet-tissue adhesion [65,84,89]. A breakthrough in this area is the development of dialdehyde-modified CMC (DCMC) and dopamine-modified chitosan (CS-DA) hydrogels for deep burn therapy. These materials not only fill irregular wound beds through their injectable, self-healing nature but also offer on-demand detachment. By applying a benign glycine (amino acid) solution, the dynamic Schiff-base bonds are competitively dissociated, allowing for the painless removal of the dressing and significantly reducing secondary trauma during dressing changes [84,89].

Similarly, CMC-graft-adipic dihydrazide (CMC-ADH) systems combined with PEG-FBA provide a versatile platform for hemostasis and wound closure [29]. These hydrogel adhesives exhibit a tissue shear stress of 54.2 kPa and can promote angiogenesis and collagen deposition while maintaining an airtight seal to prevent bacterial invasion [29,49]. The synergy between the rigid nanocellulose filler and the dynamic borate or imine linkages ensures that these adhesives remain stable under the mechanical stresses of joint movement while retaining high sensitivity for motion-sensing applications [9,38,65,70].

The integration of cellulose into hybrid adhesive systems has further enabled advanced security and monitoring functions. Photochromic self-healing adhesives have been developed by reinforcing a polylactic acid (PLA)/cellulose nanofiber (CNF) hydrogel with lanthanide aluminate nanoparticles [78]. These organic–inorganic hybrids act as “invisible” authenticating stamps that fluoresce green under 365 nm UV light, providing a durable and self-repairable solution for anti-counterfeiting in high-value products and currency.

Furthermore, the use of thiuram disulfide bonds in CNC-containing gels allows for rapid mechanical recovery under visible light, enabling the design of “smart” adhesives that can be reprocessed and recycled multiple times while maintaining high stretchability (up to 42.6 times the original length) [79]. Whether through the formation of high-density hydrogen bonds in polyurethane-CNC elastomers or through the moisture-responsive welding of siloxane-CNC composites, these adhesive platforms exemplify the transformative potential of cellulose in creating sustainable, high-performance bonding technologies [9,71,73].

### 4.4. Structural Composites: Cellulose-Mediated Healing in Cementitious Matrices

The integration of cellulose into structural composites represents a transformative approach to enhancing the durability and service life of construction materials such as concrete and mortar. In these systems, cellulose building blocks address the inherent brittleness and cracking susceptibility of cementitious matrices through three primary pathways: biological mineralization, autogenous internal curing, and encapsulated chemical repair.

A pioneering strategy in this domain involves the use of cellulose as a “smart carrier” for Microbe-Induced Calcium Carbonate Precipitation (MICP). Within the dense and highly alkaline environment of concrete (pH > 12), preserving the viability of mineral-producing bacteria is a significant challenge. Research has demonstrated that alkali-treated microcellulose fibers and porous cellulose gel beads act as effective protective scaffolds for alkaliphilic bacteria such as *Bacillus subtilis* and *Lysinibacillus sphaericus* [32,126]. These fibers serve a dual purpose: they facilitate the immobilization of bacterial spores and act as physical “bridges” across crack voids. Upon the ingress of moisture through a crack, the encapsulated microbes are activated to catalyze the hydrolysis of urea, leading to the precipitation of calcium carbonate (CaCO_3_). This biological self-healing mechanism has proven capable of sealing macroscopic cracks up to 2.5 mm in width, effectively restoring the structural integrity and durability of the infrastructure even under harsh high-temperature conditions [33,126].

Furthermore, the incorporation of nanocellulose—specifically cellulose nanofibers and nanocrystals—has emerged as a key driver for stimulating autogenous self-healing in Ultra-High Performance Fiber-Reinforced Concretes (UHPFRCs). Due to their high specific surface area and hydrophilic nature, these nanoconstituents act as internal water reservoirs that promote “internal curing” [34,125]. By releasing stored moisture during the hydration process, CNFs and CNCs facilitate the continued reaction of previously unhydrated cement particles within the crack zone. This process significantly improves the Index of Crack Sealing (ICS) and reduces the water permeability coefficient—by as much as 42% in some systems—thereby restoring the material’s water-tightness [34,125]. Recent studies have also highlighted a powerful synergy between crystalline admixtures and nanocellulose, which enhances the self-healing capacity of UHPFRC exposed to aggressive environments, such as chloride-rich geothermal waters, by refining the pore structure and hindering the transport of corrosive ions [34,35].

Finally, cellulose is employed as a sophisticated wall material in extrinsic self-healing systems for cementitious materials. Ethyl cellulose (EC) microcapsules, particularly those modified with nano-SiO_2_, offer a controllable mechanism for the sequestration and triggered release of chemical healing agents. The addition of nano-SiO_2_ increases the surface roughness and particle size of the EC microcapsules, which enhances their mechanical interlocking with the cement matrix and improves the triggering efficiency upon crack propagation [10]. These systems have achieved impressive compressive strength recovery rates (up to 151.2% under specific pre-damage conditions), demonstrating that the successful release of core materials can bridge and seal pores and cracks autonomously [10]. By combining these diverse methodologies, cellulose-based structural composites provide a robust and sustainable roadmap for the development of resilient, low-maintenance infrastructure capable of autonomous damage recovery.

## 5. Application Domains for Cellulose-Based Self-Healing Systems

The integration of self-healing functionalities into cellulose architectures has catalyzed a paradigm shift across multiple industries, transforming these materials into active, responsive systems. The diverse range of applications, spanning from soft wearable electronics to heavy-duty structural composites, leverages the multi-dynamic interactions inherent in engineered cellulose networks.

**Figure 3:**
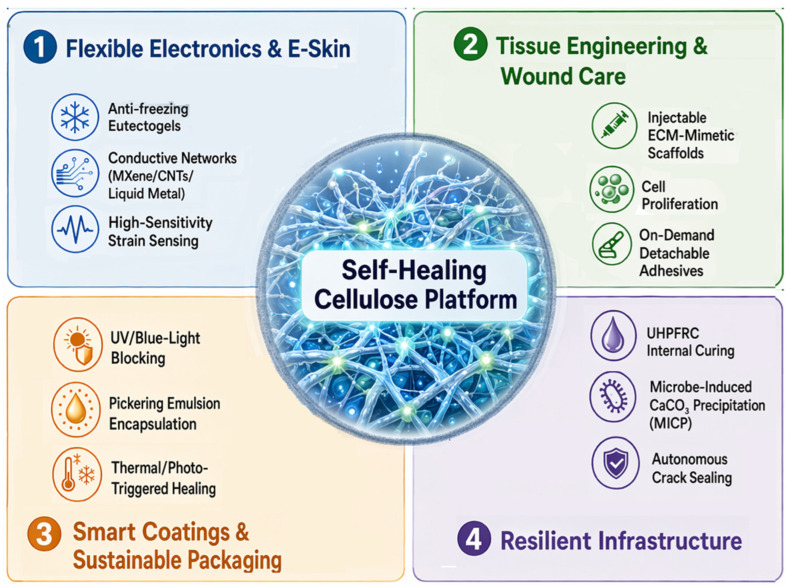
Macroscopic material formats and emerging application domains of self-healing cellulose. The central dynamic cellulose matrix can be engineered into distinct architectures tailored for specific industries: ultra-stretchable and anti-freezing sensors for wearable electronics (Quadrant 1), injectable and bioactive scaffolds for wound care and tissue engineering (Quadrant 2), UV-blocking and self-repairing films for smart packaging and coatings (Quadrant 3), and structurally resilient composites for autonomous crack sealing in civil infrastructure (Quadrant 4).

The translation of cellulose from a passive structural biopolymer into a dynamic, “living” network has catalyzed a paradigm shift across multiple high-tech industries. As illustrated in Figure 3, the macroscopic material formats of self-healing cellulose—ranging from ultra-stretchable hydrogels to robust structural composites—dictate their emerging application domains. By carefully tuning the molecular architecture and selecting the appropriate dynamic cross-links, researchers have successfully deployed cellulose platforms in four major frontiers: (1) wearable electronics and human–machine interfaces, where conductive ionogels serve as resilient e-skins; (2) biomedical engineering, utilizing injectable scaffolds for tissue regeneration; (3) smart surface technologies, offering autonomous corrosion protection and UV-shielding; and (4) civil engineering, where cellulose-mediated biocalcification and internal curing extend the lifespan of modern infrastructure. The following sections detail the specific material requirements and technological breakthroughs within each of these critical domains.

### 5.1. Wearable Electronics and Advanced Health Monitoring

The transition of cellulose from a structural biopolymer to a cornerstone of flexible electronics is driven by its ability to modulate the conductive percolation threshold and provide mechanical stability to “soft” electronic systems. Self-healing electro-conductive hydrogels (ECHs) and ionogels have emerged as the premier candidates for “electronic skin” (e-skin) applications, facilitating continuous, real-time health monitoring through biocompatible interfaces [4,24,26,27]. Within these architectures, nanocellulose (CNC, CNF, or TOCNF) performs a dual role: as a rigid mechanical reinforcement and as a high-efficiency dispersing agent that prevents the aggregation of low-dimension conductive fillers such as carbon nanotubes (CNTs), graphene, MXene, and liquid metals [8,41,55,60,64]. For example, in triple-network hydrogels, TOCNF process act as a bio-template for the in situ polymerization of polypyrrole, ensuring a stable conductive path while achieving an elongation at break of ~890% and high self-healing efficiency (98.3%) [26].

The sensing performance of these materials is characterized by exceptional sensitivity (high gauge factors) and signal linearity across broad strain windows. Cellulose-based sensors can accurately distinguish between large-scale human motions, such as the bending of fingers, wrists, or knees, and subtle physiological signals, including arterial pulse beats, respiration, and vocal cord vibrations [14,38,57,58,62,107,124,134]. Innovative designs utilizing MXene nanosheets and liquid metal nanodroplets integrated with bacterial nanocellulose (BNC) have achieved gauge factors as high as 8.09, coupled with ultra-stretchability up to 3200% [60]. This high sensitivity is maintained after structural failure because the dynamic bond network—governed by borate–diol complexes, metal–ligand coordination, or Schiff-base linkages—simultaneously restores mechanical integrity and electrical conductivity, ensuring that the sensor’s signal remains stable over thousands of loading cycles [22,59,66,95,98].

Recent breakthroughs have addressed the environmental vulnerability of traditional hydrogels, which are prone to freezing and dehydration. By transitioning to organohydrogels and eutectogels, researchers have developed sensors that maintain high flexibility and healing capacity under extreme conditions. For instance, cellulose-based eutectogels prepared via ZnCl_2_-based deep eutectic solvents (DES) exhibit anti-freezing performance at temperatures as low as −80 °C, enabling reliable sensing in polar or high-altitude environments [76,140]. The incorporation of phytic acid or zwitterionic proline into cellulose networks further enhances this environmental adaptability, effectively immobilizing water molecules to prevent ice crystal formation while maintaining a high self-healing rate (up to 92.9% at −15 °C) [25,68,75].

Furthermore, the latest generation of cellulose-based electronics features multifunctional integration, moving toward “intelligent” sensing platforms. Shape-memory functionalities enable the material to undergo programmable shape control and improve skin affinity during service [57,97,111,134]. Photoluminescence has been integrated into waterborne polyurethane-CNF composites, allowing for the visual detection of micro-cracks under UV light and facilitating timely repair to prevent catastrophic device failure [51,136]. To eliminate the need for external tapes or bandages, self-adhesive properties are introduced through mussel-inspired catechol chemistry (e.g., tannic acid or dopamine-grafted nanocellulose), ensuring robust and repeatable attachment to the human epidermis for high-fidelity signal acquisition [18,54,61,65,101,133,141]. These advancements, ranging from water-soluble and recyclable strain sensors [97] to ultra-sensitive pressure transducers for haptic sensing [69,73], establish cellulose-based self-healing materials as a versatile and sustainable platform for the future of personalized healthcare and soft robotics.

### 5.2. Biomedical Engineering: Wound Care and Tissue Regeneration

In the biomedical sector, the unique combination of high water-retention capacity, low immunogenicity, and the ease of chemical functionalization via surface hydroxyl groups makes cellulose an ideal platform for advanced clinical applications. The development of self-healing cellulose-based hydrogels has addressed the limitations of traditional static dressings by providing injectable matrices that facilitate minimally invasive delivery. By leveraging dynamic covalent linkages, such as Schiff-base reactions between dialdehyde cellulose nanocrystals (DACNCs) and carboxymethyl chitosan, these hydrogels exhibit pronounced shear-thinning behavior. This allows them to be delivered via syringe to perfectly fill the complex geometries of irregular wound beds or bone defects, where they rapidly autonomously re-form into an integrated structural piece [28,29,42,44,46,87,130,133]. For instance, host–guest interactions between β-cyclodextrin and adamantane, coupled with dynamic acylhydrazone bonds, have been successfully used to create injectable reservoirs for sustained cancer therapy, ensuring rapid structural recovery under physiological conditions [130].

A critical frontier for these materials is the management of chronic and deep-tissue wounds, particularly diabetic foot ulcers and severe burns. Cellulose hydrogels create a moist healing environment that is vital for tissue survival and re-epithelialization. Recent studies have demonstrated that dual-drug delivery systems, incorporating agents like superoxide dismutase (SOD) and epidermal growth factor (rhEGF) within a CMC-aldehyde matrix, can significantly shorten the inflammatory period and promote angiogenesis and collagen deposition in diabetic models [83,89]. A notable chemical innovation in this area is the engineering of “on-demand detachable” hydrogels. By utilizing reversible imine bonds and dopamine-modified cellulose/chitosan derivatives, researchers have developed dressings that provide robust tissue adhesion for hemostasis but can be painlessly dissolved using a benign amino acid solution (e.g., glycine), thereby eliminating the risk of secondary mechanical trauma during dressing changes [84,89]. Furthermore, the inclusion of selenium nanoparticles or marine snail peptides within these self-healing networks has shown remarkable efficacy in scavenging reactive oxygen species (ROS), thereby mitigating oxidative stress and accelerating the healing of full-thickness skin defects [31,42].

To combat the global challenge of antibiotic resistance, self-healing cellulose scaffolds are increasingly functionalized with synergistic antimicrobial and antioxidant agents. Hybrid systems utilizing bacterial cellulose nanofibers (BCNs) reinforced with silver nanoparticles, neomycin, or plant-derived polyphenols like resveratrol and gallic acid, offer broad-spectrum antibacterial activity against pathogens such as *Staphylococcus aureus* and *Escherichia coli* [16,39,45,70,86,88,92,121,141]. The dynamic borate ester or coordinate covalent bonds in these systems allow for the sustained release of these therapeutics, maintaining a sterile environment for up to 14 days [45,115]. For example, lignin-cellulose hydrogels have been shown to reduce inflammatory cell infiltration and promote M2 macrophage polarization, which is essential for transitioning from the inflammatory to the proliferative phase of healing [86].

Beyond surface wound care, the dynamic nature of these scaffolds supports the frontier of tissue engineering and cell delivery. By meticulously engineering the mechanical modulus and porosity of cellulose-based hydrogels, researchers can mimic the architecture of the natural extracellular matrix (ECM). This structural mimicry is particularly evident in cellulose-reinforced collagen or gelatin systems, which facilitate neural regeneration and preosteoblast differentiation [13,43,44,131]. These self-healing matrices perform a dual function during stem cell therapy: they protect encapsulated mesenchymal stem cells (MSCs) from lethal shear stresses during the injection process and subsequently act as a supportive scaffold that improves im-plant integrity and cell retention at the injury site [43,45]. Whether utilized for articular cartilage repair or neural stem cell differentiation, these cellulose-based platforms represent a significant leap toward sustainable and “intelligent” regenerative medicine [13,43,99].

### 5.3. Industrial Protection and Smart Surface Technologies

Cellulose-based coatings and thin films address critical challenges in industrial durability, particularly in providing autonomous defense against corrosion and electromagnetic interference (EMI). These systems leverage the high specific surface area and chemical stability of cellulose to create responsive barriers that mitigate damage in aggressive environments.

The field of corrosion inhibition has been significantly advanced by the development of cellulose-mediated extrinsic self-healing mechanisms. A primary strategy involves utilizing cellulose nanocrystal stabilized Pickering emulsions to sequester active healing agents such as linseed oil (LO) within a waterborne polyurethane (WPU) matrix. Upon mechanical injury, the CNC shell ruptures, releasing the LO which subsequently undergoes oxidative polymerization to fill surface scratches and restore the coating’s barrier properties. Research indicates that the concentration of CNCs and the size of these emulsion droplets are critical factors in optimizing the healing rate and abrasion resistance of the system [81,82].

Furthermore, ethyl cellulose (EC) and methyl cellulose (MC) are employed as robust wall materials for micro- and nanocapsules or core–shell fibers designed for localized inhibitor release. For instance, propanetriol-loaded EC microcapsules have demonstrated exceptional efficacy in protecting magnesium alloys, where the released glycerol forms a hydrophobic adsorption film at the rupture site [105]. Similarly, MC core–shell fibers fabricated via electrospinning allow for the encapsulation of oleic acid, providing high-efficiency protection for carbon steel [106]. Other systems utilize cellulose nanofibers (CNF) as “smart pathways” for the controlled release of corrosion inhibitors like dodecylamine or polyethyleneimine, where the healing response can be further modulated by the pH of the surrounding environment [12,104,144,145,146].

In the electronics sector, cellulose provides a sustainable framework for managing electromagnetic interference (EMI). By integrating flexible and highly ductile liquid metal (LM) nanodroplets with TOCNF, researchers have fabricated hydrogels that simultaneously exhibit self-healing, antibacterial properties, and high EMI shielding effectiveness (reaching up to 33.14 dB) [40]. These materials solve the interfacial compatibility issues typically associated with rigid conductive fillers. Importantly, these cellulose-LM hydrogels are fully recyclable; they can be dissolved and re-formed without compromising their shielding performance, offering a promising solution for reducing electronic waste in the wearable device industry [40,60].

The functional scope of cellulose is further extended to specialized marine applications, where biological fouling poses a severe threat to infrastructure. Cellulose-MXene double-network (DN) hydrogels, reinforced with carboxylated CNF, have demonstrated exceptional durability and long-term resistance to algal and protein adhesion in actual marine field tests. These materials exhibit an ultra-high toughness of 16,050 kJ/m^3^ and utilize photothermal-triggered self-healing to maintain their antifouling integrity for over six months [23].

The integration of vitrimer-like chemistry and dynamic covalent reshuffling has also enabled the creation of “ultrarobust” composites that bridge the gap between biodegradable materials and high-performance engineering plastics. By synergistically combining disulfide and urea bonds with hydroxyl-rich CNF, waterborne polyurethane elastomers have achieved tensile strengths of 54.4 MPa and high thermal stability [136]. Other innovations include utilizing cellulose-functionalized halloysite nanotubes (HNT-C) in epoxy coatings to facilitate transesterification-mediated healing, significantly reducing the relaxation time of the polymer chains and enhancing surface toughness [149]. Whether through UPy-modified CNCs that dissipate energy via multiple hydrogen-bonded assemblies [72] or modified siloxane elastomers capable of room-temperature healing [71], these cellulose-based architectures represent the leading edge of sustainable and resilient industrial surface technologies.

### 5.4. Sustainable Packaging and Agricultural Technologies

In the global transition toward a circular economy, cellulose-based self-healing systems are revolutionizing the design of sustainable packaging by offering high-performance functionalities that extend far beyond traditional physical barrier properties. The intrinsic renewability, biodegradability, and facile surface modification of cellulose make it the premier candidate for replacing petroleum-derived plastics, while the integration of self-healing mechanisms ensures the maintenance of package integrity and safety during the rigorous conditions of transport and storage [1,2]. A significant breakthrough in this domain is the development of multi-functional hydrogel packaging films derived from nanocellulose (CNF) and polyvinyl alcohol, integrated with advanced functional fillers such as ZIF-8-embedded curcumin (Cur@ZIF-8) [132]. These hybrid films utilize the dense hydrogen-bonding network of cellulose to achieve a 49.2% improvement in water vapor barrier properties and a 193.5% increase in tensile strength compared to control systems. Crucially, the dynamic borate ester and hydrogen bonding within the matrix allow for autonomous repair of mechanical punctures, while the ammonia-sensitive nature of the curcumin enables the packaging to function as a real-time freshness indicator. In practical packaging tests, these cellulose-based films inhibited microbial growth and spoilage, effectively extending the shelf life of protein-rich foods, such as fish, to nine days [132].

Beyond structural integrity, cellulose architectures are being engineered for advanced light management and photodegradation protection within the packaging and high-tech building sectors. Composite films fabricated from microfibrillated cellulose (MFC) and benzoxazine resins (BR) achieve a unique synergy between mechanical robustness (tensile strength of 142.99 MPa) and thermal-healing capabilities, where surface creases and notches can be repaired via simple heating with a hairdryer [102]. These films provide a gentler lighting environment by blocking 98.65% of ultraviolet (UV) radiation and 68.85% of blue light, which is essential for preserving the nutritional value and color stability of light-sensitive consumer goods. Similarly, self-healing waterborne polyurethane (WPU) coatings, facilitated by CNC-stabilized Pickering emulsions of linseed oil, offer a transparent yet high-haze solution for lamp covers and glass surfaces, ensuring uniform light distribution and privacy protection without sacrificing high light transmission [81].

The implementation of sophisticated encapsulation technologies further enhances the environmental resilience of these sustainable materials. Novel cellulose-silica hybrid microcapsules have been developed using one-step emulsion-solvent diffusion, where hydrophobic coupling reagents and UV absorbers are grafted onto the capsule shell [148]. These coatings exhibit self-repairable superhydrophobicity and UV protection, maintaining their functional performance even after seven cycles of chemical immersion or eight cycles of mechanical abrasion. In the agricultural sector, the structural versatility of self-healing cellulose hydrogels is being explored for the construction of superabsorbent systems designed for moisture retention and the controlled release of nutrients, particularly in arid regions where they can minimize runoff and improve water use efficiency [2]. By combining autonomous structural recovery with smart sensing, high-efficiency light management, and advanced barrier properties, cellulose-based platforms are establishing a new standard for intelligent, bio-based packaging solutions that drastically reduce food waste and environmental degradation [81,102,132,148].

### 5.5. Civil Engineering and Infrastructure

The structural longevity of modern infrastructure is being significantly enhanced through the integration of cellulose building blocks into cementitious matrices, such as concrete and mortar. In the high-alkaline environment characteristic of construction materials (pH > 12), cellulose serves as a versatile “smart” component that addresses the inherent brittleness and susceptibility of concrete to macroscopic cracking. One of the most technologically advanced pathways is the utilization of cellulose as a protective carrier for Microbe-Induced Calcium Carbonate Precipitation (MICP). Research has demonstrated that alkali-treated microcellulose fibers and porous cellulose gel beads provide a sustainable and cost-effective habitat for mineral-producing, alkaliphilic bacteria such as Bacillus subtilis or *Lysinibacillus sphaericus* [32,33,126]. From a chemical perspective, the cellulose matrix facilitates the immobilization of bacterial spores and protects them from the intense mechanical stresses of the mixing process and the chemical aggression of the cement paste. Once a crack propagates, the ingress of moisture and oxygen activates the encapsulated microbes, which catalyze the hydrolysis of urea to trigger the precipitation of calcite (CaCO_3_). This biological self-healing mechanism has demonstrated a remarkable capacity to autonomously seal cracks up to 2.5 mm in width, effectively restoring the water-tightness and split tensile strength of the concrete by up to 25% [33,126]. Furthermore, these cellulose fibers act as physical “bridges” across crack voids, increasing the localized availability of bacteria and ensuring a more uniform mineral deposition [32].

Beyond biological repair, cellulose plays a critical role in promoting autogenous self-healing and internal curing, particularly in Ultra-High-Performance Fiber-Reinforced Concretes (UHPFRCs). Due to their extreme hydrophilicity, high specific surface area, and high aspect ratio, cellulose nanofibers and nanocrystals function as internal water reservoirs that modulate hydration kinetics [34,35,125]. During the cracking process, these nanoconstituents release stored moisture to facilitate the continued hydration of previously unreacted cement and supplementary cementitious particles, such as slag, within the crack zone. This “internal curing” effect significantly improves the Index of Crack Sealing (ICS) and has been shown to reduce the water permeability coefficient by 42% [34,125]. Recent evidence suggests that the presence of CNCs and CNFs favors the formation of portlandite and leads to a significantly denser cement paste with a refined pore structure, which is vital for infrastructure exposed to aggressive chemical environments like chloride-rich marine conditions or geothermal waters [34,35]. In such environments, the nanocellulose-mediated healing significantly hinders the transport of corrosive ions, thereby preserving the structural integrity of the steel reinforcement.

Additionally, cellulose is utilized as a sophisticated wall material for extrinsic self-healing systems. The development of ethyl cellulose (EC) microcapsules modified with nano-SiO_2_ allows for the sequestration and controlled release of chemical healing agents. The organic–inorganic hybridization of EC with nano-SiO_2_ increases the surface roughness and particle size of the capsules, which enhances their mechanical interlocking with the cement matrix and improves the triggering efficiency upon crack propagation [10]. These systems have achieved impressive compressive strength recovery rates of 151.2% in damaged mortar specimens, demonstrating that the successful release of core materials can effectively bridge both microscopic pores and macroscopic cracks autonomously [10]. By synergizing biological mineralization, nanostructural pore refinement, and encapsulated chemical repair, cellulose-based strategies offer a robust and sustainable roadmap for constructing resilient, low-maintenance infrastructure with a significantly reduced carbon and environmental footprint.

The material formats discussed in this section provide the essential structural and chemical platform for self-repair, but their utility is ultimately defined by the requirements of the end-use environment. The transition from a self-healing hydrogel or structural composite to a functional device—such as a high-fidelity wearable sensor, a bioactive wound dressing, or a resilient infrastructure component—requires a precise calibration of the material’s mechanical, electrical, and biological properties. The following section evaluates these architectures within the context of their specific application domains, demonstrating how the synergy between cellulose matrices and their healing mechanisms is optimized to meet the distinct challenges of human health monitoring, civil engineering, and industrial protection.

## 6. Commercial Translation and the Global Patent Landscape

The transition of cellulose-based self-healing materials from academic proof-of-concept to highly protected intellectual property marks a critical milestone in their industrial maturation. A comprehensive analysis of recent global patents reveals a strategic utilization of cellulose derivatives and nanostructures to engineer autonomous repair mechanisms. These patented innovations span a vast array of high-value sectors, leveraging both the intrinsic dynamic covalent chemistry of cellulose and its extrinsic capacity as a robust encapsulating agent.

Historically, the incorporation of cellulose derivatives into protective and adhesive matrices was explored as early as the 1980s and 1990s (e.g., early patents by Saint-Gobain and 3M). However, a chronological analysis of the current dataset reveals an exponential surge in patent filings occurring post-2015, with a dense concentration of active and pending applications filed between 2020 and 2025, as can be seen in Table 1. This timeline perfectly mirrors the global scientific shift toward nanocellulose extraction, the push for circular economies, and the maturation of dynamic covalent chemistry. The anticipated expiration dates of these patents—many stretching well into the late 2030s and early 2040s—indicate that industries are laying the groundwork for long-term commercial monopolies over the next two decades.

A demographic analysis of the table reveals a distinct bipolar dominance in the commercialization of cellulose-based self-healing materials, driven primarily by the United States and China, alongside highly specialized contributions from Europe and the Asia-Pacific region.

China exhibits a massive and aggressive patenting strategy, particularly in the last five to seven years. Chinese patents overwhelmingly dominate the domains of soft electronics, hydrogel sensors, and civil engineering infrastructure. Notably, the majority of Chinese patents are held by major academic institutions (e.g., Peking University, South China University of Technology, Nanjing Forestry University), indicating heavily state-funded R&D pipelines aimed at rapid tech-transfer to industry. Similarly, South Korea shows strong, targeted intellectual property in 3D printing and eco-friendly construction materials, reflecting its highly advanced electronics and chemical sectors.

The United States maintains a patent portfolio characterized by high-tech, specialized applications. US patents are heavily clustered in advanced biomedical engineering (e.g., targeted drug delivery and tissue scaffolding led by MIT and Johns Hopkins) and high-performance industrial coatings (driven by corporate entities like HRL Laboratories, 3M, and Baker Hughes).

European nations, while producing a smaller volume of patents compared to the US and China, demonstrate a strong focus on fundamental green chemistry, biodegradability, and sophisticated composite engineering. Countries like Denmark, Spain, and France are pioneering patents in sustainable waterproof membranes, bio-covalent hydrogels, and nanocomposite optimization (e.g., Nanocore ApS, CNRS). Furthermore, Europe shows a high degree of cross-border and cross-institutional collaboration, exemplified by joint patents filed between Danish universities, Spanish research centers, and US institutions (e.g., Novo Nordisk collaborating with MIT).

In the biomedical sector, the patent landscape is heavily focused on the rheological modulation of cellulose to create biocompatible, self-healing architectures for tissue engineering and pharmaceutical delivery. Patents have secured methodologies for in situ forming nanofiber-hydrogel composites that utilize cellulose esters (such as cellulose acetate and cellulose butyrate) combined with thickeners like hydroxypropyl methylcellulose (HPMC) to autonomously conform to and repair soft tissue defects [151,161]. The dynamic covalent capacity of cellulose is extensively claimed; for instance, the synthesis of self-healing hydrogels via Schiff-base reactions between formylated methylcellulose and PEGylated chitosan is patented specifically for the encapsulation and sustained release of exosomes, accelerating skin wound repair [159]. Broad claims also cover dynamic covalent hydrogels utilizing carboxymethyl cellulose and hyaluronic acid for versatile pharmaceutical applications [155], as well as transition metal thiolate-crosslinked networks incorporating CMC for highly elastic, self-assembling medical materials [156].

Furthermore, cellulose derivatives are indispensable in the design of sophisticated autonomous administration devices. Groundbreaking patents describe “self-righting” gastrointestinal capsules engineered to orient themselves against mucosal tissue and inject pharmaceutical agents. These mechanical capsules rely on cellulose derivatives (e.g., HPMC, cellulose acetate phthalate, ethyl cellulose) to form precisely controlled fluid gates, moisture-responsive degradable supports, and mucoadhesive tissue-interfacing components [153,154]. Other biomedical patents include the use of cellulose in sacrificial matrices to fabricate intricate vascular network preforms for engineered tissues [158], the formulation of shear-thinning, self-healing networks using hydrophobically modified HPMC for targeted drug delivery [152], and flexible, multi-layered ultrasound-transparent gel pads utilizing nanocellulose for self-healing diagnostic interfaces [150].

The intersection of cellulose chemistry and flexible electronics is a rapidly expanding patent domain, focusing on highly stretchable, electro-sensitive, and robust hydrogels. Intellectual property here exploits the synergistic effects of dynamic borate ester bonds, metal–ligand coordination, and hydrogen bonding. Patents describe ultrafast self-healing polysaccharide-based strain sensors that combine soluble cellulose derivatives with polyvinyl alcohol and borax, enabling rapid underwater and atmospheric healing for real-time human motion monitoring [165,202]. To enhance conductivity and mechanical resilience, patents claim multi-network hydrogels formulated via gamma-ray or electron beam irradiation, utilizing cellulose alongside metal ions (Fe^3+^, Al^3+^) to achieve extraordinary adhesive forces and electro-sensitivity [164,167]. Similarly, the integration of plant polyphenols, tannic acid, and dopamine with nanocellulose or CMC is patented to yield highly conductive, antibacterial, and self-adhesive hydrogels suitable for bio-electrodes and flexible capacitors [157,160,166].

The structural role of cellulose is also claimed in advanced manufacturing and core electronic components. For instance, cellulose nanofibers (CNF) combined with cerium ions have been patented for self-healing ion-conductive gels tailored specifically for 3D printing of tactile sensing and artificial skin devices [163]. In high-tech hardware, novel self-healing field-effect transistors (FETs) rely on cellulose as a flexible, self-repairing backing layer to support semi-conducting elongated nanostructures [168]. Energy storage innovations include patented multifunctional polymer binders for lithium-ion battery anodes, wherein CMC is crosslinked with conductive and self-healing polymers (like dopamine and polypyrrole) to accommodate the massive volume expansion of silicon-graphite electrodes during charge cycling [169]. Additionally, the integration of TEMPO-oxidized nanocellulose with carbon nanomaterials (CNTs) and polyacrylic acid has been patented to form robust, self-healing intelligent gels [162].

The protective coatings industry exhibits a dense patent portfolio centered on utilizing cellulose for both intrinsic dynamic bonding and extrinsic microencapsulation. Foundational patents have long recognized the utility of cellulose plastics in polyurethane-based adhesive coatings for laminated safety glass [171], which has evolved into modern claims for scratch-resistant, self-healing polyurethane networks utilizing cellulose derivatives as nonionic surfactants and rheology modifiers [175]. In contemporary intrinsic systems, patents highlight the functionalization of cellulose nanocrystals with furan rings to engage in thermally reversible Diels–Alder reactions within waterborne polyurethane matrices, yielding sustainable coatings with enhanced gas barrier and self-repair properties [183,184].

For extrinsic self-healing, ethyl cellulose (EC) is the commercial standard for encapsulating active agents. Patents extensively cover the fabrication of EC microcapsules and nanocapsules loaded with linseed oil or proprietary healing agents, dispersed within acrylic or polyurethane matrices, which rupture to seal scratches autonomously [178]. This encapsulation strategy is deployed in multiphase coatings [177], self-healing coatings derived from recycled polymer blends [182], and specialized coatings for the oil and gas sector [176]. Beyond physical repair, cellulose is crucial in advanced surface functionalization. Patents protect superhydrophobic, omniphobic, and slippery coatings where cellulose nanocrystals and derivatives act as pore-forming additives or functional fillers to maintain repellent properties after severe abrasion [173,174,180,187]. Specialized optical and safety coatings include reversible, light-responsive ionomer systems utilizing metal-ion reduction in cellulose matrices [179], as well as high-efficiency fire-retardant paints that combine graphene oxide with functionalized cellulosics (e.g., HEC, HPMC) to provide active fire-warning alongside structural self-healing [172,181,185].

In civil engineering and heavy industry, patents aggressively scale the protective and delivery capabilities of cellulose for infrastructure repair. A major commercial trajectory involves the microencapsulation of biological or chemical healing agents to repair microscopic cracks in concrete and coal mine ventilation seals. Patents describe the formulation of sophisticated core–shell microcapsules where microcrystalline cellulose (MCC), HPMC, and ethyl cellulose serve as the porous core matrix and the stress-sensitive wall material. These capsules protect mineral-producing bacterial spores or swelling agents (like sulphoaluminate cement) until crack propagation triggers their release, facilitating sustainable biocalcification or chemical expansion [190,191,195]. Cellulose ethers are also claimed as critical water-soluble polymers in leakage self-repairing waterproof mortars [193], eco-friendly cement compositions boasting exceptional chemical resistance [194], and systems utilizing CO_2_ nanobubbles and calcium hydrate for deep-crack carbonation healing [196].

Cellulose is also revolutionizing flexible infrastructure and extractive operations. Patented technologies for self-healing asphalt pavements utilize natural wood cellulose fibers as flexible reinforcements to minimize low-temperature cracking [192], while other claims protect hollow fibers with a cellulose acetate-plasticized shell designed to slowly release asphalt rejuvenating agents into the bitumen matrix [189]. In the geomaterials sector, extruded self-supporting waterproofing membranes incorporate sodium carboxymethyl cellulose as a super water-absorbent polymer alongside thermoplastic polyolefins [200]. Furthermore, in subterranean oil and gas operations, hydrophilically modified cellulose derivatives are patented as relative permeability modifiers to autonomously degrade and heal filter cakes in open-hole well completions [201], and as crucial viscosity enhancers and erosion-resistant fibers for packing solid materials and proppants in underground formations [200]. Finally, the synthesis of macroscopic nanocomposites using cavitation methods to anchor cellulose acetate or nitrate to nanotubes ensures highly dispersed, ultra-strong, and self-repairing bulk materials for heavy industrial use [197,199].

### Challenges in the Commercialization of Cellulose-Based Self-Healing Materials

Recent advances in cellulose-based self-healing materials have expanded rapidly, yet their transition from laboratory-scale research to commercial implementation continues to face major multifaceted bottlenecks. Scaling these systems from controlled synthesis environments to industrial manufacturing requires overcoming key challenges associated with cost efficiency, long-term stability, and the lack of standardized performance evaluation protocols.

A primary barrier to industrial adoption is the disparity between academic synthesis and manufacturing feasibility. Many current laboratory protocols rely on sophisticated, multi-step chemical modifications, such as extensive periodate oxidation followed by complex grafting procedures or the use of rare, high-purity catalysts. Although these methods are highly effective at achieving rapid healing in a research setting, they are often prohibitively expensive and energy-intensive for commodity-scale production. Industrial scaling demands “one-pot,” solvent-free, or aqueous-based synthetic pathways that minimize secondary waste and energy consumption. Achieving high-performance sensors through simplified processes remains a significant challenge, as the current reliance on specialized chemical crosslinkers inherently limits the economic viability of these smart materials in mass-market applications.

The gap between laboratory-grade performance and real-world durability is perhaps the most significant constraint for cellulose-based self-healing platforms. Most academic self-healing hydrogels are optimized for a stable, benign laboratory environment (25 °C, 50% relative humidity). In contrast, commercial applications—particularly in the construction and electronics sectors—require robustness against intense UV radiation, extreme temperature fluctuations, and aggressive cyclic mechanical fatigue. Currently, many hydrogel-based sensors struggle to maintain signal stability over extended service lives due to phenomena like signal drift, progressive dehydration, or freeze–thaw fatigue.

To illustrate this translational gap, Table 2 delineates the performance requirements necessary for industrial viability versus the limitations currently observed in laboratory-scale cellulose materials.

Another critical barrier limiting the commercialization of cellulose self-healing systems is the absence of standardized testing protocols. Currently, there is a lack of universal guidelines for measuring “self-healing efficiency.” Parameters such as crack width, specific damage location, healing duration, temperature, and environmental conditions vary significantly across studies. These metrics are inherently sensitive to the experimental configuration, including crack geometry, the contact pressure applied to the fracture surfaces, and the environmental history of the sample.

This lack of standardization makes it impossible for industrial partners to certify these materials for safety-critical applications, such as load-bearing infrastructure or medical devices. Consequently, there is an urgent need for the cellulose community to adopt universal guidelines for damage recovery assessment. Establishing rigorous, standardized protocols for crack width, recovery kinetics, and environmental stability testing is essential for providing the regulatory confidence required for industrial adoption and ensuring that self-healing performance is reported in a manner that is both reproducible and industry-relevant.

Finally, the path to commercialization must align with the circular bioeconomy. Incorporating self-healing features often necessitates the blending of cellulose with non-biodegradable synthetic polymers, such as polyacrylamide or certain polyurethane derivatives, which complicates end-of-life recycling. Future industrial-scale designs must prioritize “closed-loop” systems, where the dynamic covalent bonds that enable self-healing also facilitate on-demand, triggered depolymerization. By shifting toward fully bio-based materials that combine self-repair with inherent recyclability, the industry can move beyond the current laboratory focus on temporary functionality and toward a paradigm of long-term, resilient, and environmentally compliant smart materials.

## 7. Conclusions and Future Perspectives

The transition toward sustainable, resilient, and intelligent materials has positioned cellulose at the forefront of polymer science. However, in the self-healing subdomain, a critical evaluation of its macromolecular architecture reveals a fundamental paradox: native cellulose is intrinsically non-ideal for autonomous self-healing. The very characteristics that provide cellulose with its renowned structural integrity—its highly ordered crystalline domains and dense, rigid network of intra- and intermolecular hydrogen bonds—severely restrict polymer chain movement. Because self-healing relies fundamentally on chain mobility (reptation) and the dynamic reshuffling of bonds across a damaged interface, unmodified cellulose cannot heal autonomously [203].

Nevertheless, the true value of cellulose lies in its chemical versatility and structural hierarchy when utilized in combinations and hybrid systems. By serving as a functional co-component, cellulose resolves the classic polymer physics dilemma between mechanical robustness and healing efficiency. Through physical nanoscale extraction (yielding CNCs and CNFs) or chemical functionalization (producing derivatives like CMC, HEC, and dialdehyde cellulose), it is transformed from a rigid barrier into a dynamic building block. In these hybrid systems, cellulose acts as a reinforcing filler, a multi-site crosslinking node, or a stabilizing scaffold for dynamic covalent and supramolecular networks.

Because of this combinatory approach, cellulose is successfully utilized in a diverse array of material formats, each tailored to specific operational environments. In highly hydrated or solvated states, it forms advanced hydrogels, organohydrogels, and eutectogels that are the backbone of wearable flexible electronics, e-skin, and injectable biomedical matrices. In solid-state applications, it is engineered into elastomers, transparent films, and robust industrial coatings. Furthermore, exploiting its hydrophobic derivatives (like ethyl cellulose) and macro-fibrous forms, it is formulated into microcapsules and microbial carriers for extrinsic self-healing in heavy-duty structural composites, such as asphalt and concrete.

A comparative analysis between current laboratory research and the global patent landscape reveals a distinct translational gap. In academic laboratories, research is heavily focused on highly sophisticated intrinsic self-healing mechanisms. Scientists are engineering complex, multi-dynamic networks—such as simultaneously coupling Schiff-base reactions with metal–ligand coordination and host–guest interactions—to achieve ultra-fast healing kinetics, extreme stretchability (exceeding 4000%), and multi-stimuli responsiveness (e.g., photo-thermal, magnetic, and pH triggers). In the patented industrial sector, however, the focus shifts toward scalability, cost-effectiveness, and reliability. The patent landscape is heavily dominated by extrinsic healing strategies—specifically, the use of ethyl cellulose microcapsules to deliver liquid healing agents (epoxies, linseed oil, or corrosion inhibitors) and bacteria into concrete or protective coatings. Academic research continues to push the boundaries of “smart” dynamic gels. However, industry relies primarily on the stable, proven film-forming and encapsulating properties of traditional cellulose derivatives to protect large-scale infrastructure and industrial surfaces.

Despite the significant advances made in cellulose-based self-healing materials, translating these laboratory-scale chemical breakthroughs into heavy-duty commercial applications remains the primary frontier of the field. As demonstrated by our comprehensive analysis of the global patent landscape, a clear dichotomy persists: while academic research is heavily concentrated on intrinsic, multi-dynamic soft-matter systems (such as stretchable hydrogels and biomedical scaffolds), industrial intellectual property is predominantly focused on extrinsic, encapsulation-based strategies for protective coatings and civil infrastructure. This translational gap underscores several unresolved challenges that must be addressed to facilitate industrial adoption. Key technological bottlenecks include ensuring long-term environmental stability under harsh cyclic loading, establishing standardized protocols for healing efficiency measurement, and the development of cost-effective, green synthesis pathways that avoid hazardous catalysts. Future research must prioritize “closed-loop” material design—where self-healing functionality is coupled with on-demand recyclability—to fully align these intelligent platforms with the requirements of a circular bioeconomy. Addressing these issues will not only bridge the divide between fundamental chemistry and industrial practice but will also define the commercial viability of next-generation, bio-derived, and resilient smart materials.

The progress in cellulose-based self-healing materials is undeniable, but several critical challenges must be addressed to facilitate their widespread commercialization and ensure they meet the criteria for true sustainability:

- Green Synthesis and Modification: Many current protocols for modifying cellulose (e.g., periodate oxidation or grafting with synthetic polymers) still rely on toxic solvents, heavy metal catalysts, or complex, energy-intensive purification steps. Future research must prioritize green chemistry approaches, such as using deep eutectic solvents (DES) or aqueous-based, one-pot syntheses, to ensure the entire lifecycle of the material is environmentally benign.
- Advanced Manufacturing and 4D Printing: While 3D printing of cellulose-based inks has made significant strides, achieving high structural resolution without compromising the dynamic mobility required for self-healing remains a critical hurdle. Future research should prioritize the convergence of additive manufacturing with stimuli-responsive “4D printing.” By leveraging the inherent responsiveness of cellulose networks to external triggers (such as pH, moisture, or temperature), researchers can create complex, shape-morphing architectures that autonomously adapt to their environment or repair deep-seated mechanical damage over time. Optimizing ink rheology for “one-pot” printable self-healing systems is essential for transitioning from static components to personalized, integrated devices in the soft robotics and biomedical sectors.
- Long-Term Environmental Stability: Self-healing hydrogels perform exceptionally well in controlled laboratory environments, but their real-world application is limited by dehydration, freezing, or swelling. Advancing the design of eutectogels and integrating anti-fatigue mechanisms that can withstand thousands of mechanical cycles over years—not just days—is essential for the commercialization of wearable electronics and bio-implants.
- Standardization of Healing Metrics: Currently, the literature suffers from a lack of standardized testing protocols for self-healing efficiency. Establishing universal guidelines for measuring damage recovery (e.g., specifying crack size, healing time, temperature, and required rest periods) is necessary to accurately compare different cellulose composites.
- End-of-Life and Recyclability: Finally, incorporating self-healing features often involves blending cellulose with non-biodegradable synthetic polymers (like polyacrylamide or polyurethane). Future designs must focus on fully bio-based, closed-loop systems where dynamic covalent bonds not only provide self-healing but also enable on-demand depolymerization, allowing the materials to be fully recycled or biodegraded at the end of their service life.

Looking toward the next decade, the frontier of cellulose-based self-healing materials will be defined by the convergence of green chemistry with advanced digital and manufacturing technologies. A critical emerging direction is the integration of Artificial Intelligence (AI) and Machine Learning (ML) in materials discovery. AI-guided predictive modeling can significantly accelerate the optimization of self-healing networks by simulating dynamic bond kinetics, predicting the ideal ratio of nanocellulose fillers, and mapping the stiffness–extensibility trade-off without the need for exhaustive trial-and-error experimentation.

In conclusion, while cellulose alone cannot heal, its integration into dynamic architectures has proven to be one of the most promising avenues in modern materials science. By continuing to bridge the gap between complex laboratory innovations and scalable industrial patents, cellulose-based self-healing systems will play a foundational role in the next generation of sustainable, resilient, and intelligent technologies.

## Figures and Tables

**Table 1:** Categorized patent landscape of cellulose-based self-healing materials.

Application Domain	Patent Title	Type of Cellulose Described	Applicant/Assignee	Country of Origin	Year (File/Pub)	Expiration	Status	References
**1. Biomedical, Health & Tissue Engineering**	A Flexible, Stretchable, Adhesive Multilayered Gel Pad Displaying Self-Healing Capacity…	Cellulose, Methacrylated Cellulose, Nano-cellulose	Univ Danmarks Tekniske, Univ Basque Country	Denmark/Spain	Filed 2024	N/A	Pending	[150]
	In Situ Forming Composite Material For Tissue Restoration	CA, CAP, CAB, CP, CB, CPB, CDA, CTA, MC, HPC, HEC, HPMC	Univ Johns Hopkins	USA	N/A	N/A	Discontinued	[151]
	Shear-thinning self-healing networks	HPMC, CMC, CNC, CNF	Massachusetts Institute of Technology	USA	N/A	2035	Active	[152]
	Self-healing system, method, and related components	HPMC, HPC, HEC, EC, CAP, CAT, CMC, MC	Brigham and Womens Hospital, MIT	USA	N/A	2038	Active	[153]
	Systems and methods for liquid injection	HPMC, HPC, HEC, EC, CAP, CAT, Cellulose	Novo Nordisk AS, Brigham and Womens Hospital, MIT	Denmark/USA	Pub 2025	N/A	Pending	[154]
	Dynamic covalent hydrogels, their precursors and applications	Cellulose, CMC, HMC, HPC, MC, Methoxy Cellulose	CNRS, Univ de Nantes, INSERM	France	N/A	2041	Active	[155]
	Self-repairing material and method for its preparation	CMC, Calcium CMC, Sodium HPC	Fundacion Cidetec	Spain	N/A	2032	Active	[156]
	A kind of preparation method of plant polyphenol nano-cellulose…	Nanocellulose, MCC	Fujian Agriculture and Forestry Univ	China	N/A	2038	Active	[157]
	Fabrication of a vascular system using sacrificial structures	Cellulose	Cornell University	USA	N/A	2031	Active	[158]
	Self-healing hydrogel encapsulating exosomes, preparation method…	Formaldehyde Methylcellulose (Aldehyde MC)	Tongji University	China	N/A	2039	Expired—Fee	[159]
	Preparation method of multi-responsiveness self-healing self-adhesion hydrogel	Bacterial Cellulose (BC)	Harbin Institute of Technology Shenzhen	China	N/A	2041	Expired—Fee	[160]
	Bandage-free antibacterial and anti-adhesion nanofiber dressing and preparation method thereof	Ethyl cellulose	Hubei Chunshuo Technology Partnership Enterprise LP	China	2021	2041	Active	[161]
**2. Soft Electronics, Sensors & Energy Storage**	Preparation method and application method of TEMPO nano-cellulose-polyacrylic acid gel	TEMPO Nano-cellulose (TOCNF)	Nanjing Forestry University	China	N/A	2038	Active	[162]
	Self-healing ion conductive gel composition for 3D printing	Cellulose Nanofibers (CNF)	Jeong Gyeong-un, Shin Woo-hyeon	South Korea	N/A	2040	Active	[163]
	A kind of self-healing flexible hydrogel electrosensitive material…	Sodium CMC	Yanshan University	China	N/A	2038	Active	[164]
	Ultrafast self-healing polysaccharide-based hydrogel strain sensor…	Soluble derivatives (CMC, HEC, HPC, HPMC)	East China Normal University	China	N/A	2040	Active	[165]
	Self-healing hydrogel of cellulose-dopamine-polymer composite material…	Cellulose (Cotton linters, corncobs), CMC, HEC	Technical Institute of Physics and Chemistry of CAS	China	N/A	2038	Active	[166]
	A kind of high-adhesion conductive self-healing hydrogel and its preparation…	Cellulose derivatives	Peking University	China	N/A	2037	Active	[167]
	Multi-functional field effect transistor with intrinsic self-healing properties	Cellulose	Technion R&D Foundation Ltd.	Israel	N/A	2039	Active	[168]
	Multifunctional polymer binder for anode and method of producing same	Sodium CMC	Sicona Battery Technologies Pty Ltd.	Australia	N/A	2040	Active	[169]
	Preparation method of metal ion coordinated natural polymer/polyacrylic acid…	HEC	Southeast University	China	N/A	2037	Active	[170]
**3. Smart Coatings, Films & Surface Protection**	Polyurethane-based adhesive coating or film…	Cellulose plastic, CA, CAB	Saint-Gobain Vitrage	France	Filed 1986	N/A	Granted	[171]
	Biomimetic, moldable, self-assembled cellulose silica-based trimeric hydrogels…	HEC, MC	Leland Stanford Junior University	USA	N/A	2038	Active	[172]
	Self-healing, self-cleaning omniphobic composition, related articles…	CNC, Cellulose, Lignocellulose	Michigan State University MSU	USA	N/A	2040	Active	[173]
	Self-healing laminate composition, related articles and related methods	CNC	Michigan State University (MSU)	USA	N/A	2040	Active	[174]
	Reactive two-part polyurethane compositions and optionally self-healable…	CA, CAB, CAP	3M	USA	Pub 1998	N/A	Expired	[175]
	Self-healing coatings for oil and gas applications	CMC, HEC	Baker Hughes Holdings LLC	USA	N/A	2036	Active	[176]
	Multiphase coatings with separated functional particles…	Cellulosic polymers, Cellulose Hydrogels, MC, CMC, HEC, HPC	HRL Laboratories LLC	USA	N/A	2038	Active	[177]
	Reversible, chemically or environmentally responsive polymers, and coatings…	Cellulose, Modified Cellulose, CMC, HEC, HPC, MC	HRL Laboratories LLC	USA	N/A	2036	Active	[178]
	Microcapsule, self-healing coating material forming composition…	Cellulose	Korea Construction Living Environment Testing Inst.	South Korea	N/A	2031	Expired—Fee	[179]
	Reinforced composites with repellent and slippery properties	Cellulose or a derivative	Battelle Memorial Institute Inc	USA	N/A	2037	Active	[180]
	High-efficiency flame retardant coating with fire warning and self-healing functions…	HPC, HPMC, HEC	South China University of Technology SCUT	China	N/A	2038	Active	[181]
	Self-healing coatings from recycled polymer blends	HEC, MC, CMC	Empire Technology Development LLC	USA	N/A	2033	Expired—Fee	[182]
	A kind of Cellulose nanocrystal enhancing selfreparing water-base polyurethane…	CNC	Wuhu Wanlong New Material Co., Ltd.	China	Pub 2019	N/A	Pending	[183]
	A bio-based waterborne polyurethane material self-repairing performance enhancer…	CMC	Central South Univ, Zhuzhou Times New Material	China	N/A	2043	Active	[184]
	A kind of self-healing type steel structure fireproof coating…	Cellulose, HEC	Hubei Polymeric Polymer Material Co.	China	N/A	2036	Active	[185]
	Corrosion-resistant coating for marine concrete…	EC, Hydroxymethyl Cellulose, HEC, CMC	Guilin University of Technology	China	N/A	2023	Ceased	[186]
	Durable superhydrophobic surfaces	CA, EC	Univ of Michigan, US Dept of Air Force	USA	N/A	2038	Active	[187]
**4. Civil Engineering & Resilient Infrastructure**	Self-supporting, synthetic polymer waterproof membrane with self-healing ability	Sodium CMC	ATARFIL SL	Spain	N/A	2036	Active	[188]
	Self-healing and rejuvenating materials for asphalt mixtures	CA	Louisiana State University	USA	N/A	2040	Active	[189]
	Microcapsule of sustainable self-healing coal mine ventilation sealing…	HPMC, MCC	Shandong University of Science and Technology	China	Pub 2021	N/A	Abandoned	[190]
	Microcapsule for self-healing concrete and preparation method thereof…	MCC, EC	Shenzhen University	China	Pub 2018	N/A	Abandoned	[191]
	Crack self-healing bituminous concrete and preparation method thereof	Natural wood cellulose fiber	Zhengzhou University	China	N/A	2032	Active	[192]
	Leakage self-healing waterproof mortar and preparation method thereof	Cellulose Ether	Zhejiang Luban Building Materials, ZJUT	China	N/A	2036	Active	[193]
	Self-healing eco-friendly cement mortar composition for repairing structure…	CA	Reflash Tech, KSC Construction, Solution E&C	South Korea	N/A	2037	Active	[194]
	Intelligent trigger type concrete micro-crack self-repairing microcapsule…	EC, HPMC, MCC	Research Institute of Highway Ministry of Transport	China	N/A	2041	Active	[195]
	Method of repairing and reinforcing cross-section of concrete structure…	MC, Hydroxymethyl Cellulose, CMC, EC, HEC, Carboxyethyl Cellulose, HPC	Eco E&C Co., Ltd.	South Korea	N/A	2039	Active	[196]
**5. Industrial Materials & Extractive Industries**	Composite Materials And Processes For The Preparation Thereof	CA, Cellulose Nitrate, CMC, HEC	Nanocore Aps	Denmark	Filed 2023	N/A	Pending	[197]
	Polymer Latex For The Preparation Of An Elastomeric Film…	CMC, HEC, HPC, Modified celluloses	Synthomer Sdn Bhd	Malaysia/UK	Pub 2021	N/A	Application	[198]
	Repair And Optimization of Nanocomposite Materials	Cellulose Acetates, Cellulose Propionates, Cellulose Butyrates, MC	Nanocore Aps	Denmark	Filed 2025	N/A	Pending	[199]
	Method of packing solid materials during underground treatment operations	CMC, CMHEC, HEC, CA	Lubrizol Corporate	USA	Pub 2019	N/A	Active	[200]
	Self healing filter-cake removal system for open hole completions	Cellulose	Halliburton Energy Services Inc	USA	N/A	2030	Not-in-force	[201]
	Preparation method of environment-friendly fast self-healing hydrogel	Nanocellulose, Cellulose powder	Central South University of Forestry and Technology	China	N/A	2039	Active	[202]

Abbreviations: CMC = Carboxymethyl Cellulose; HEC = Hydroxyethyl Cellulose; HPC = Hydroxypropyl Cellulose; HPMC = Hydroxypropyl Methylcellulose; MC = Methyl Cellulose; EC = Ethyl Cellulose; CA/CAB/CAP = Cellulose Acetate/Cellulose Acetate Butyrate/Cellulose Acetate Propionate; CNC/CNF/TOCNF = Cellulose Nanocrystals/Cellulose Nanofibers/TEMPO-Oxidized Cellulose Nanofibers; MCC = Microcrystalline Cellulose.

**Table 2:** Industrial requirements vs. laboratory limitations in cellulose-based systems.

Application Field	Current Laboratory Limitations	Target Industrial Requirements
Biomedical	High cost of chemical crosslinkers; insufficient long-term chronic toxicity data.	Sterility, low-cost scalability, rapid absorption, standardized biocompatibility testing.
Construction	Limited healing range of micro-cracks; prohibitively high cost of microbial carriers.	Cost-effectiveness (per ton), 50-year service life, compatibility with bulk concrete.
Electronics	Signal drift after repeated self-healing cycles; slow evaporative loss of hydrogel solvents.	10,000+ cycles of fatigue resistance, anti-drying/anti-freezing, high signal sensitivity.

## Data Availability

No new data were produced.
